# Neutrophil depletion at the early stage of Japanese encephalitis virus infection affects CD8+ T cell infiltration into the mouse brain and causes severe encephalitis

**DOI:** 10.3389/fimmu.2025.1748085

**Published:** 2026-01-21

**Authors:** Rohit Soni, Prasanjit Jena, B. Kusuma, Arup Banerjee

**Affiliations:** Laboratory of Virology, Regional Centre for Biotechnology, National Capital Region Biotech Science Cluster, Faridabad, India

**Keywords:** BBB, CD8, CXCR4, Japanese encephalitis virus, neutrophils

## Abstract

Neutrophils have been reported to have protective and detrimental functions in viral infections. However, the role of neutrophils remains unexplored in Japanese encephalitis virus (JEV) infection. In this study, we elucidated the dynamics of neutrophils and their influence on immune cell recruitment in subclinical and severe encephalitis in mouse models. Further, we depleted neutrophils from 3–4 week-old C57BL/6 mice using mAb1A8 (anti-Ly6G) antibody and studied their association with inflammation, viral replication, immune cell infiltration and disease outcome. We observed that an increase in JEV replication is associated with increased infiltration of neutrophils in the spleen and brain. Further studies confirmed that depletion of neutrophils at an early stage of JEV infection reduced CD8 abundance in the infected brain and accelerated death in mice. We also observed that inhibition of the CXCL12-CXCR4 signalling axis by antagonist AMD3100 reduced CD8 abundance in the brain and augmented inflammasome activation, leading to fatal encephalitis. Reduced CXCR4 levels in the spleen and blood of CD8+T cells correlated with enhanced Granzyme B level, indicating CD8 cells differentiated more into effector phenotypes in neutrophil-depleted mice. Furthermore, CD8 depletion delayed the death of mice infected with a sublethal strain compared to neutrophil-depleted mice, suggesting that neutrophils play a vital role in the early restriction of viral replication, whereas CD8 is essential later in clearing the virus. Taken together, our study sheds new light on the role of neutrophils in the pathogenic mechanisms of JEV encephalitis and highlights the importance of neutrophils and CD8 cells associated with disease outcomes.

## Introduction

The host’s innate immunity plays a significant role in the initial response against the viral infection. Despite patrolling the peripheral innate immune cells, the neurotropic virus evades the immune barrier and enters the brain, giving rise to encephalitis syndrome. Japanese Encephalitis Virus (JEV), a neurotropic Flavivirus, causes encephalitis symptoms that affect children and adults in tropical and subtropical countries, including India, resulting in morbidity and mortality (1). The host immune system’s components work cooperatively with viral variables to establish the severity of the infection (2). JEV can penetrate the central nervous system (CNS), resulting in various pathogenic effects. Due to a breach in BBB function, peripheral immune cells infiltrate and cause bystander damage in the brain (3). However, antigen-specific lymphocytes can accumulate in the CNS and assist in defence by decreasing or clearing the invading viral infection (4). The clinically apparent JE results in high case fatality (~ 20–30%) and severe neurological deficits in 30–50% of survivors. However, the pathogenic mechanisms of fatal and subclinical JEV encephalitis are not entirely understood.

Myeloid cells, e.g., neutrophils, have been implicated in contributing to both host defence and disease in response to viral infection of the CNS (5, 6). Neutrophils are first-line responders to infections and are recruited to target tissues through the action of chemoattractant molecules, such as chemokines. Neutrophils are crucial for controlling bacterial and fungal infections (7, 8), but their role in viral infections has been understudied. Studies using the WNV virus show that neutrophils have a variety of functions during infection. They first seem to protect against WNV infection, but eventually appear detrimental (9). Neutrophils have become essential parts of the regulatory and effector circuits of the innate and adaptive immune systems. Neutrophils communicate with other adaptive immune system cells, such as macrophages, dendritic cells, and others, directly or through soluble mediators (10). The extracellular chemoattractant gradient, essential for drawing neutrophils to the infection site, guides the migration of these leukocytes to the site of infection. When JHM, a strain of Mouse Hepatitis virus (JHMV), infects the CNS, neutrophils are quickly recruited from the bone marrow and into circulation (11, 12). This is most likely due to a sharp rise in the production of the neutrophil chemoattractant. Neutrophils promote effector CD8+ T cell migration into the influenza-infected trachea by releasing chemokine trails. Neutrophils have been implicated in both protective and pathological responses following virus infections. We hypothesize that differences in the ability to recruit and activate neutrophils in peripheral organs and the brain may affect peripheral immune cell infiltration in infected brains and modulate disease outcomes.

Understanding the pathophysiological mechanisms of encephalitis has been challenging due to inadequate animal models of neurological disease. We previously isolated mice adapted to the JEV virus (JEV-S1; #OK257214 source: C57BL/6 pups ‘ brains, and JEV-S3; # OK257215 source: 3–4 week C57BL/6 adolescent mice brains), which showed proper encephalitis signs in 3 to 4-week-old mice (13). We also previously reported that, in C57BL/6 mice, intraperitoneal (IP) inoculation with the JEV-S1 isolates resulted in subclinical disease. In contrast, inoculation of the mouse-adapted strain JEV-S3 caused fatal encephalitis (13). Using these mouse-adapted isolates in this study, we elucidated the dynamics of neutrophils and their influence on immune cell recruitment in subclinical and severe encephalitis in mouse models. We also studied their association with inflammation, viral replication, and disease outcome. Our study demonstrated that neutrophil modulation significantly impacts CD8 cell infiltration in the brain and affects disease outcome together.

## Materials and methods

### Ethics and biosafety statements

Animal experiments were approved by the Regional Centre for Biotechnology Institute (RCB) Animal Ethics Committee (approval no. RCB/IAEC/2020/082). The Ministry of Environment and Forestry, Government of India, Committee for Control and Supervision of Experiments on Animals criteria were strictly followed when handling the animals. They were kept in an environment with a 12-hour light/dark cycle, consistent temperature, and an ad libitum food and water supply. The Institutional Biosafety Committee (approval no. RCB/IBSC/21-22/334) duly approved the protocol. All *in vitro* and animal experiments were carried out strictly under BSL-2 containment, per the Biosafety guidelines issued by the Department of Biotechnology, Ministry of Science & Technology, Government of India.

### Virus isolates

In our previous study (13), we generated different stages of mouse-adapted JEV virus through sequential passages of porcine kidney (PS) cell-grown, sucrose gradient-purified JEV (P20078 strain) into pups to adolescent mice (3–4 weeks old), followed by virus amplification in pups. Briefly, in stage 1, sequential passages of porcine kidney (PS) cell-grown, sucrose gradient-purified JEV (P20778 strain; GenBank accession no AF080251) were passaged four times in ten-day-old pups’ brains. Pups developed paralysis symptoms at 72–96 hpi. Brain tissue was collected to prepare a virus suspension in MEM. The virus strain isolated at the end of Stage 1 was designated as JEV-S1.

Then, in Stage 2, these JEV-S1 were again passaged multiple times in 3–4 week-old mice via intravenous injection. After multiple passages, when injected intravenously, the Stage 2 virus showed consistently high titre in the mice’s brains. The Stage 2 virus, i.e., JEV-S2, was then reamplified in pups’ brains, and the recovered virus was referred to as the Stage 3 virus or JEV-S3. Mice adapted to the JEV virus (JEV-S1; GenBank accession no. OK257214, and JEV-S3; GenBank accession no. OK257215, source: 3–4 week C57BL/6 adolescent mice brains), previously described (13), were used in this study. JEV-S1 and JEV-S3 exhibited differences in virulence in adolescent mice (3–4 week old mice) when injected intraperitoneally (IP).

### Cell culture and viral infection

The Huh7, Neuro2a (N2a) obtained from the cell repository at the National Centre for Cell Sciences, Pune, India, and EA.hy926 cells (# ATCC-CRL-2922) were cultured in Dulbecco’s modified Eagle’s medium (DMEM) (11960; Invitrogen, Carlsbad, CA, USA) with 10% FBS. Cell lines were maintained at 37°C in the CO2 incubator. Cells were seeded at 80% confluence and infected with JEV-S1 & JEV-S3 at a multiplicity of infection (MOI) =1 for one h; cells were harvested at 6, 12, 24 & 36 hpi for lysate preparation in Tris-HCl buffer (pH 7.5) with 1mM phenylmethylsulfonylfluoride (P7626-250MG; Sigma-Aldrich, Darmstadt, Germany).

### RNA extraction and qRT-PCR

Total RNA was extracted from the tissue samples using TRIzol reagent (15-596-018; Invitrogen, Carlsbad, CA, USA). In two steps, a GoScriptTM Reverse Transcription System from (A5003; Promega, Madison, WI, USA) was used to create cDNA. Gene expression was measured by qRT-PCR in triplicate using SYBR green DNA-binding fluorescent dye (SYBR premix Ex Taq, RR420; Takara, Otsu, Shiga, Japan) on a Quanta Studio 6 Flex Real-Time PCR System (Applied Biosystems, Foster City, CA, USA). The genes were amplified using primers listed in **Table 1**. Relative gene expression in JEV-infected cells was estimated by measuring the delta Ct value with mock-infected cells as the reference and Gapdh as an internal control.

**Table 1:** List of primers for gene expression analysis.

Gene	Forward sequence	Reverse sequence
GAPDH	CGTCCCGTAGACAAAATGGT	TTGATGGCAACAATCTCCAC
JEV	TTACTCAGCGCAAGTAGGAGCGT CTCAAG	ATGCCGTGCTTGAGGGGGACG
NLRP3	TGCTCTTCACTGCTATCAAGCCCT	ACAAGCCTTTGCTCCAGACCCTAT
ASC	CTTGTCAGGGGATGAACTCAAAA	GCCATACGACTCCAGATAGTAGC
IL-1B	TCCAAGAAAGGACGAACATTCG	TGAGGACATCTCCCACGTCAA
Caspae-1	ACAAACACCCTGACAAACCAC	CACTGCGTTCAGCATTGTTAAA
IL-18	GCTTGAATCTAAATTATCAGTC	GAAGATTCAAATTGCATCTTAT

### Immunoblotting

A protein lysate was extracted by homogenizing mouse brain tissue in a Tris-HCl buffer (pH 7.5) containing 1 mM phenylmethylsulfonylfluoride (P7626-250MG; Sigma-Aldrich, Darmstadt, Germany). We used the bicinchoninic acid test to calculate the protein concentration (A55860; Pierce, Rockford, IL, USA). Equal amounts of protein (30 μg) were separated by 10% SDS-PAGE and transferred to a PVDF (polyvinylidene difluoride) membrane. The membrane was probed with either rabbit anti-GAPDH antibody (1:10,000) (GTX100118; GeneTex, Irvine, CA, USA) or rabbit polyclonal antibody against JEV NS3 protein (1:15,000) (GTX125868; GeneTex, Irvine, CA, USA), ASC (1;1000) (D2W8U, CST, Massachusetts, USA), Cleaved Caspase-1 (1:1000) (Asp297, CST, Massachusetts, USA), Caspase-1 (1:1000) (#2225, CST, Massachusetts, USA), Claudin-5 antibody (1:500) (sc-374221, Santa Cruz, Biotechnology, Dallas, TX, USA), Luminol reagent (sc-2048; Santa Cruz, Biotechnology, Dallas, TX, USA) was used for chemiluminescence detection, and a ChemiDocTM XRS System (Bio-Rad, Hercules, CA, USA) was utilised to view the signal.

### Virus titration by plaque-forming assay

Vero (African green monkey) cells obtained from the National Centre for Cell Science, Pune, India, were maintained at 37°C with 5% CO2 in minimal Eagle’s medium (11095080; Invitrogen, Carlsbad, CA, USA) with 10% FBS, 1% penicillin-streptomycin, and glutamine. We used Vero cells with 60% confluence per well for virus titration. Cells were washed with 1x PBS, then incubated with 10-fold serially diluted virus stock prepared in serum-free Eagle’s minimal essential medium (MEM) for one hour, and incubated at 37°C with gentle rocking. Post incubation, cells were washed with 1xPBS and overlaid with 2 ml of agarose type VII (A0701; Sigma-Aldrich, Darmstadt, Germany) in MEM, prepared by mixing a 1:1 ratio of 2x minimal Eagle’s medium (HiMedia) with 10% FBS and a 2% solution of low-melting agarose. After 5 days, cells were fixed with 3.7% formaldehyde (252549-25ml; Sigma-Aldrich, Darmstadt, Germany). The overlay plugs were removed, and cells were stained with 1% crystal violet (C6158-50G; Sigma-Aldrich, Darmstadt, Germany) for 5 min. Plaques were counted, and the virus titer was calculated using the formula: virus titer (pfu/ml) = average pfu/volume of infection (ml) x dilution factor.

### Immunofluorescence assay

Neuro2a cells were seeded on the coverslips in a 24-well plate at a confluency of 80%, then the cells were infected with JEV-S1 & JEV-S3 at MOI = 1 for one h. After 24 h, cells were fixed and permeabilized with 4% paraformaldehyde (PFA) (158127-5G; Sigma-Aldrich, Darmstadt, Germany) for 15 min at RT, then blocked with 1% BSA (A9418-5G; Sigma-Aldrich, Darmstadt, Germany) for one h at room temperature. After blocking, Primary antibody staining for JEV NS3 protein (1:1500) (GTX125868; GeneTex, Irvine, CA, USA) was done for one hour at room temperature (RT). Cells were washed multiple times with PBS, Alexa Fluor 488, goat anti-mouse (1:600) (A11029; Invitrogen, Carlsbad, CA, USA) was added for one h at RT, Following multiple wash with 1X PBS, Coverslips were then mounted, and the nucleus was stained with 4′,6-diamidine-2′- pheynylindole dihydrochloride (DAPI) (D1306; Invitrogen, Carlsbad, CA, USA). Images were acquired at 40x magnification using a confocal microscope (Leica SP5 Laser Confocal Microscope) and analyzed using ImageJ software.

### JE clinical sign scoring

Mice injected with JEV were monitored daily for body weight, and clinical scores were given based on the following signs. # 1 for piloerection; # 2, for body stiffening, piloerection; # 3, for body stiffening, piloerection, restriction of movement; # 4, for body stiffening, piloerection, limitation of movement, hind limb paralysis; #5, for body stiffening, piloerection, restriction of movement, hind limb paralysis, whole-body tremor.

### Virus entry assay

The Huh7 and Neuro2a cells were seeded in 12-well plates at around 0.35*10^6 10^6^ cells per well. Cells at 80% confluency were infected on ice with JEV-S1 & JEV-S3 at an MOI of 1. Then, after one hour, the wash was given with ice-cold PBS, and then complete media was added and incubated for one hour at 37°C. After one hour, cells were trypsinized, the 1x PBS wash was given, and TRIzol was added to the pellet for further RNA isolation.

### *In Vivo* neutrophil depletion

Anti-Ly6G (clone 1A8, #BP0075-1) and anti-rat Kappa immunoglobulin (clone MAR18.5, # BE0122) were purchased from Bio X Cell, USA, and injected intraperitoneally following the schemes and dosage that are presented in the figures or legends.

### BBB disruption study

The Evans Blue extravasation assay was performed to study the change in BBB permeability. C57BL/6 mice (3–4 weeks old) were injected intraperitoneally (IP) with 100 μL 1% Evans Blue dye (E2129, Sigma-Aldrich, Darmstadt, Germany) prepared in sterile PBS. After one hour of dye incubation, the mice were euthanised by carbon dioxide inhalation (30-70%) in consultation with a veterinarian, and the image was captured. Blood was collected before euthanasia.

### *In vivo* depletion of CD8+ T-cells

Anti-mouse CD8a (clone 53.6-7, #BP0004-1), anti-rat Kappa immunoglobulin (clone MAR18.5, # BE0122), and corresponding were purchased from Bio X Cell, USA, and injected intraperitoneally following the schemes and dosage that are presented in the figures or legends.

### Immune cell isolation from mouse blood and brain for flow cytometry analysis

Blood was collected from mice through retro-orbital bleeding and added to RBC lysis buffer (00-4300-54; Invitrogen, Carlsbad, CA, USA) for 15 minutes at room temperature. The mixture was then centrifuged at 1500 rpm for 10 minutes, and the pellet was resuspended in 1x PBS. Mice were anaesthetised with ketamine (100 mg/kg), xylazine (4 mg/kg) and perfused with ice-cold PBS. The brain was removed and homogenized with a Dounce homogenizer (D8938; Sigma-Aldrich, Saint Louis, MO, USA) in ice-cold Hanks balanced salt solution (HBSS) buffer (Sigma-Aldrich, Saint Louis, MO, USA). Brain homogenate was resuspended to prepare 30% isotonic Percoll (17089102; Cytiva, Marlborough, MA 01752, USA), which was overlayed on 70% isotonic Percoll. Then, a gradient was centrifuged at 600g for 25 min at 20°C. Mononuclear cells were collected from a 30%/70% interface and washed with PBS. To identify monocytes, granulocytes and Lymphocytes, isolated mononuclear cells from blood and brain were first pre-incubated with anti-mouse CD16/32 antibody (101302; BioLegend, San Diego, CA) for 10 mins at room temperature and then simultaneously stained with fluorochrome-conjugated antibodies CD11b (101211, BioLegend, San Diego, CA), Ly6G (127606; BioLegend, San Diego, CA), Ly6C (128018, BioLegend, San Diego, CA), CD3 (100204; BioLegend, San Diego, CA), CD4 (10043; BioLegend, San Diego, CA), CD8 (100737; BioLegend, San Diego, CA), anti-Gr1 (553123; BD Pharmingen, Qume Drive, San Jose, CA 95131, United States). Cells were then rinsed with 1X PBS and run on the BD FACS Verse (BD Biosciences, San Jose, CA). Data were analyzed using FlowJo v10.

### Administration of AMD3100

AMD3100 Octahydrochloride hydrate (A5602; Sigma-Aldrich, St Louis, MO), a specific CXCR4 antagonist, was injected intraperitoneally at (4 mg/kg) or vehicle (PBS) and was administered twice daily for 5 days. AMD3100 was dissolved in PBS, and a PBS control injection was administered to the control mice.

### Chemokine measurement using ELISA

The serum and brain lysate from infected mice (JEV-S1 & JEV-S3) were used to detect CXCL12 (E-EL-M3046; Elabscience, Texas, USA), MIF-1 (EM0440, Fine Test, Broadway Street, US), CXCL10 (EM0004, Fine Test, Broadway Street, US), and CCL2 (EM0135, Fine Test, Broadway Street, US). According to the manufacturer’s guidelines, 30 μ g of equivalent protein concentration samples were diluted with assay buffer and added to the ELISA plate. The observation was taken at 560 nm (SpectraMax i3x Multi-mode Detection Platform). Values were calculated by subtracting the value of the blank and quantified against the standard.

### Intracellular and surface staining of CD8 T cells from Blood and splenocytes

Intracellular staining was performed to characterize T cell subsets. Briefly, 2 × 10^6^ blood cells and splenocytes were seeded per well in a flat-bottom 96-well plate and stimulated for four h with phorbol 12-myristate 13-acetate (PMA; 50 ng/mL; Sigma-Aldrich) and ionomycin (1 µg/mL; Sigma-Aldrich) in the presence of the protein transport inhibitor monensin (#554724, GolgiStop, BD Biosciences). Cells were first stained with surface markers for 15–20 min at room temperature in PBS containing 1% FBS, then fixed with Cytofix and permeabilized using the Fixation/Permeabilization Solution Kit (#554714, BD Biosciences). Intracellular staining was performed in Perm/Wash buffer with the following fluorochrome-conjugated antibodies: CD8^+^ T cells were stained with CD8a (APC; #200081-U; Clone S18018E), Granzyme B (FITC; #396404; Clone QA18A28) as previously described (PMC10333319; PMID: 37949319; PMID: 28993609). Stained cells were acquired on a BD FACS Symphony™ instrument (BD Biosciences) using BD FACS Suite software version 1.0.6 and analyzed with FlowJo software version VX (Tree Star, San Carlos, CA, USA).

### Statistical analysis

Statistical analysis was performed using Prism version 8.0.2 (GraphPad Software, San Diego, CA, USA). Values are expressed as the Mean ± SD, with a significance level of *P* < 0.05. Mean survival time (MST) was analyzed by plotting the Kaplan–Meier curve using a log-rank test.

## Results

### JEV-S3 replicates faster than JEV-S1 in *in vitro* and mouse models and is associated with increased inflammasome activation in the brain

**Figure 1:**
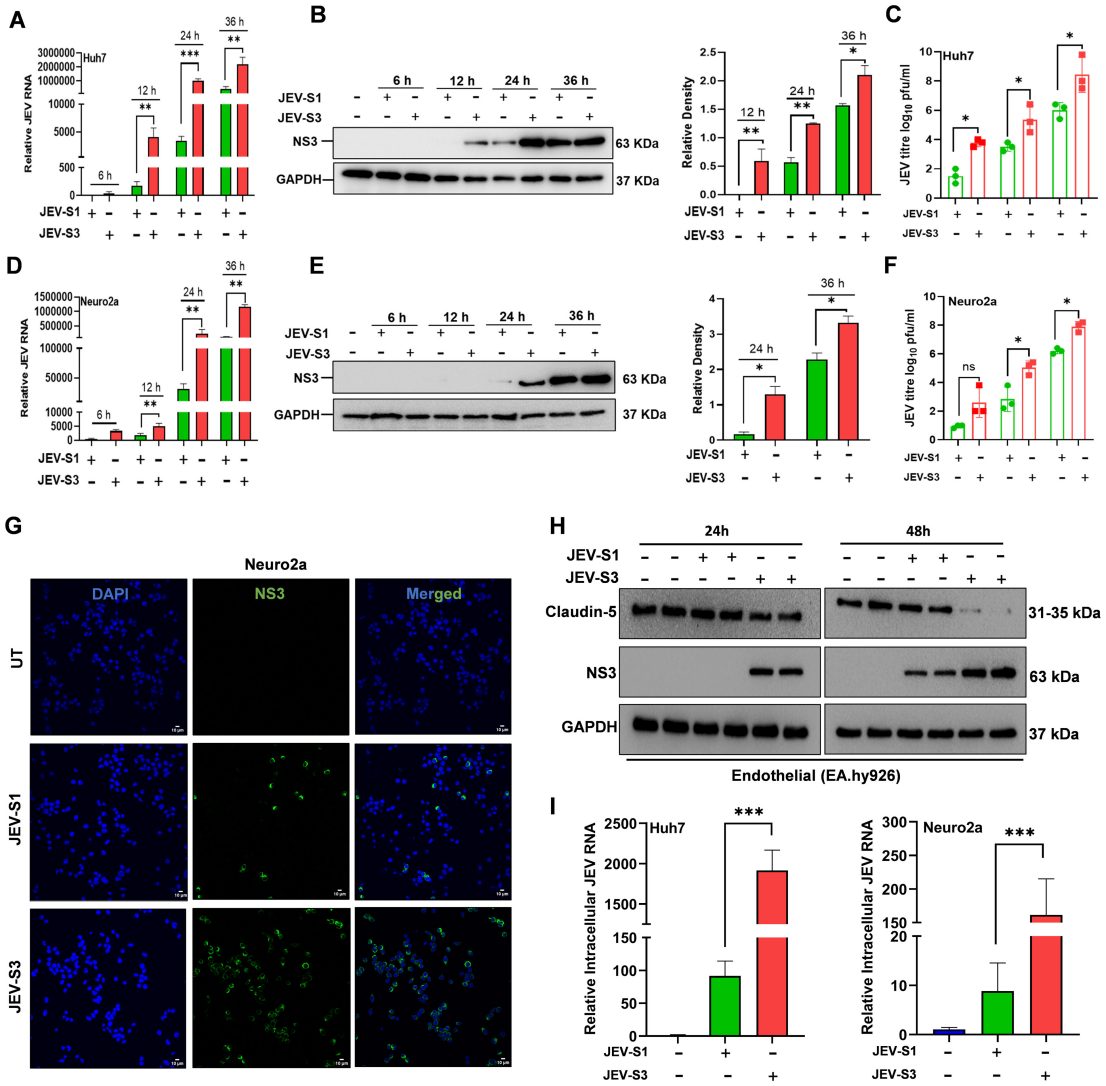
JEV-S3 replicates faster than JEV-S1 in non-neuronal and neuronal cells. **(A)** Huh7 and **(D)** Neuro2a cells were infected with JEV-S1 and JEV-S3 at an MOI of 1 and harvested at 6, 12, 24, and 36 hpi. With qRT-PCR, we quantified the JEV mRNA. The fold changes of JEV were related to the internal control of GAPDH. Data in all bar graphs are presented as mean ± SD. P*-*values were calculated using two-tailed unpaired Student’s t-test; *P<0.05; **P<0.01; ***P<0.001. Three independent experiments were conducted, each experiment set with a triplicate. **(B)** Huh7 and **(E)** Neuro2a cells were infected with JEV-S1 and JEV-S3 at an MOI of 1, and the cells were harvested at 6, 12, 24, and 36 hpi. The expression levels of NS3 and GAPDH were detected by immunoblotting. The corresponding molecular weight of the protein is shown on the right side. The relative densities were calculated by measuring the band intensity using ImageJ. Two independent experiments were performed. **(C, F)** Vero cells were infected with cell supernatant collected from Huh7 and Neuro2a at different time points to quantify virus yield by plaque assay. **(G)** Neuro2a cells infected with JEV-S1 and JEV-S3 at an MOI of 1 were stained for JEV antigen (NS3, green) and DAPI (Nucleus, blue) at 24 hpi. Images were captured using a confocal microscope (magnification 40X, Scale bar=.10 μm) **(H)**. EA.hy926 cells were infected with JEV-S1 and JEV-S3 at an MOI of 1, and the cells were harvested at 24 and 48 hpi. The expression levels of NS3, Claudin-5, and GAPDH were detected by immunoblotting. **(I)** Neuro2a and Huh7 cells were infected with JEV-S1 and JEV-S3 at MOI = 1 on ice for one hour, followed by incubation for one hour at 37°C. Cells were harvested after one hour of incubation, and total RNA was used for viral RNA detection by RT-PCR. Data shown in the bar graph are presented as mean ± SD. P*-values* are calculated using an unpaired Student’s t-test; *P<0.05; **P<0.01.

In our previous study, we generated different stages of mouse-adapted JEV virus strain through sequential passages of porcine kidney (PS) cell-grown, sucrose gradient-purified JEV (P20078 strain) into pups (Stage 1) to adolescent mice (3–4 weeks old) (Stage 2), followed by virus reamplification in pups (Stage 3). The virus isolated at the end of Stage 1 and Stage 3 was designated as JEV-S1 and JEV-S3, respectively. To begin with, we looked into the replication kinetics of JEV-S1 and JEV-S3 in three cell lines (Huh7, Neuro2a (N2a), and EA.hy926 cells) of varying tissue origins (hepatocytes, neuroblastoma, and endothelial cells, respectively). We performed a time course analysis of virus infection with JEV-S1 and JEV-S3 in Huh7 and N2a cells by measuring intracellular viral RNA, protein, and infectious virus particles released in the supernatant using the qRT-PCR, Western blot, and plaque assay methods. As shown in **Figures 1A–F**, JEV-S3 RNA and NS3 protein levels were significantly higher at 12, 24, and 36 h post-infection (hpi) compared to JEV-S1 in both human hepatoma (Huh7) and mouse neuroblastoma (Neuro2a, N2a) cells. The same is also reflected in the virus titre. About 2 log fold differences were observed between JEV-S3 and JEV-S1 in Huh7 and N2a cells at 24 hpi (**Figures 1C, F**). We further checked replication kinetics using an immunofluorescence method described in the Materials and Methods. In N2a cells, we observed that the number of infected cells is higher in the case of JEV-S3 than JEV-S1 (**Figure 1G**). Similar results were also observed on endothelial cells (EA.hy926) infected with JEV-S1 and JEV-S3 viruses. JEV-S3 replicated faster in endothelial cells as viral protein expression was evident within 24 hpi, whereas viral protein expression was noted only at 48 hpi with JEV-S1 (**Figure 1H**).

There is also a difference observed at the entry level. We infected Huh7 and N2a at MOI = 1 for JEV-S1 & JEV-S3 for one hour on ice, followed by one hour of incubation at 37°C, then measured the amount of intracellular viral RNA internalized into cells. The internalized JEV-S3 viral RNA level was higher than JEV-S1 (**Figure 1I**). Thus, our study showed that JEV-S1 and JEV-S3 isolates exhibited differences in viral entry, replication, and virus release in neuronal and non-neuronal cells.

Our previous study also demonstrated that JEV-S1 and JEV-S3 exhibited differences in virulence in adolescent mice. The mean survival time (MST) for the mice inoculated with 10^8^ pfu of JEV-S1 and JEV-S3 was 17 and 7.5 days, respectively (13). We used these virus isolates, JEV-S1 & JEV-S3, to model mild and severe JEV symptoms. Using this mouse model, we studied the differences in peripheral immune cells mediated response to illuminate the pathogenic mechanisms resulting in lethal and mild encephalitic disease. We further studied replication kinetics and infiltrated immune cell levels in different organs of mice infected with JEV-S1 and JEV-S3 isolates.

**Figure 2:**
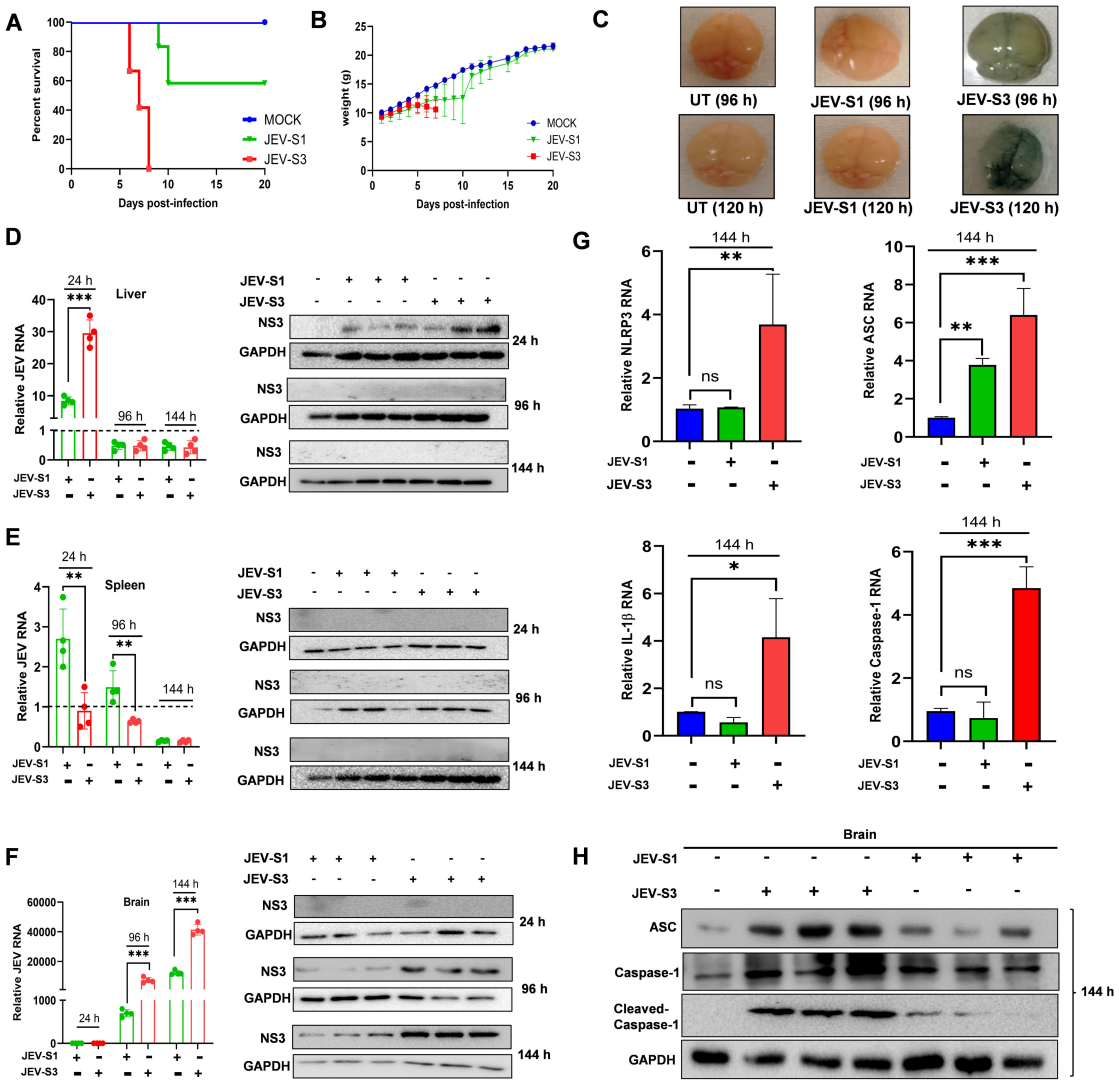
Replication Kinetics of JEV-S1 and JEV-S3 in C57BL/6 mice. C57BL/6 mice (3–4 weeks old) were injected with 10^7^ pfu/ml of JEV-S1 and JEV-S3 through intraperitoneal injection. **(A)** Kaplan-Meier survival curve of JEV-S1 and JEV-S3 infected C57BL/6 (n=9 in each group). **(B)** The change in the body weight of JEV-S1 and JEV-S3-infected mice. **(C)** Evans Blue dye (100µl) was injected IP on 96 and 120 hours post-infection (hpi). The mice’s brains were harvested one hour later, and the image was captured. **(D)** Liver, **(E)** Spleen, and **(F)** Brains of C57BL/6 mice (n=4) were harvested at 24, 96, and 144 h and assessed for viral load and neuronal damage. The JEV mRNA was quantified by qRT-PCR. The fold changes of JEV were normalized to the internal control GAPDH, and the expression levels of NS3 and GAPDH were detected by immunoblotting. **(G)** qRT-PCR method used to quantify the NLRP3 inflammasome genes in the brain at 144 h in JEV-S1 and JEV-S3, and the fold changes of genes were normalized to the internal control GAPDH. **(H)** The expression levels of ASC, Caspase-1, Cleaved Caspase-1, and GAPDH were detected in the infected mice’s brain by immunoblotting. ns P > 0.05, * P ≤ 0.05, ** P ≤ 0.01. *** P ≤ 0.001.

As shown in **Figure 2A**, C57BL/6 (3–4 weeks old) mice infected with JEV-S1 did not develop severe clinical signs at an early point. JEV-S1-infected mice showed delayed symptoms; about 40% of mice died later, and the rest survived. This starkly contrasts with what happens after JEV-S3 infection in the same strain of mice. Mice succumbed to encephalitis disease within 5–7 days after JEV-S3 infection; signs of illness (e.g., weight loss, tremors & neurological signs) began at 5 days post-infection (dpi), while in the case of JEV-S1, most of the mice survived (**Figure 2A**). The body weight of the JEV-S3-infected mice decreased sharply after day 5 compared to JEV-S1. In contrast, in the case of JEV-S1, after initial weight restrictions, mice regained weight from day 10 (**Figure 2B**). We studied the BBB integrity in mice following virus infection. Evans Blue dye was injected in live mice through the intraperitoneal (IP) route on days 4 & 5 post-infection, and the mouse brain was harvested one h later. The brain from the virus-infected JEV-S3 mice showed Evans blue uptake at days 4 & 5 pi, suggesting a BBB breach, while the dye is not detected in the JEV-S1 infected brain, suggesting a BBB is intact in the case of JEV-S1 (**Figure 2C**). This was also evident from endothelial cell data (**Figure 1H**), where JEV-S3 infection significantly reduced tight junction protein (Claudin-5) in endothelial cells, confirming modulation of tight junction protein expression by JEV-S3. Thus, our study further confirmed that JEV-S1 and JEV-S3 behave differently in mice regarding virulence, even with the same number of infectious particles when given for the infection. Further, we infected mice with a dose of 10^7^ pfu/ml (JEV-S1 and JEV-S3) and monitored the viral spread at 24, 96, and 144 hpi in different tissues, including liver, spleen, and brain, using qRT-PCR, Western blots (**Figures 2D–F**). Within 24 hpi, viral RNA and protein NS3 (JEV-S1 and S3) were transiently detectable in the liver but remained undetectable at 96 and 144 hpi in the liver and spleen of mice (**Figures 2D, E**). However, we found detectable viral RNA and protein in the brains of JEV-S1 and JEV-S3 infected mice as early as 96 hpi (**Figure 2F**). A relatively lesser amount of JEV-S1 RNA and proteins were detectable in brains at early time points of infection. Also, compared to JEV-S3, JEV-S1 in the brain replicated slowly, similar to data observed in our *in vitro* studies (**Figure 1**).

It was reported earlier that tick-borne viruses can cross the blood-brain barrier via a transcellular pathway without compromising the integrity of the monolayer of endothelial cells (14). Since JEV-S1 is detectable in the brain in lesser amounts, we checked if JEV-S1 could infect endothelial cells to enter the brain. We observed that endothelial cells are sensitive to JEV-S3 infection with high JEV NS3 protein expression within 48 hpi and a significantly reduced level of tight junction protein, suggesting barrier damage. However, JEV-S1 infected endothelial cells but replicated at a slower rate than JEV-S3, with considerably less viral protein expression, and no change in tight junction protein was observed (**Figure 1H**). It could be possible that JEV-S1 probably enters the brain without compromising BBB integrity via other means, as reported earlier (14, 15). Further study may be needed to shed more light on this issue.

Next, we investigated whether the difference in replication capacity also affects brain inflammation. Inflammasome activation could correlate with the virulence (16). JEV triggers inflammasome activation in infected mice brains (17). To understand if JEV-S1 and JEV-S3 induce NLRP3 activation differentially, we infected the mice with JEV-S1 and JEV-S3 (10^7^ pfu/ml) and harvested the brains at 144 hpi. We then carried out qRT-PCR and Western blot to measure different genes associated with inflammasome formation. Our results showed a robust increase in NLRP3, ASC, IL-1β, and caspase-1 levels in JEV-S3-infected mice brains, respectively, compared to JEV-S1 (**Figure 2G**). The activation and processing of Caspase-1 are key events during inflammasome activation. We performed the Western blot analysis to determine the changes in the protein levels of ASC, Caspase-1, and Cleaved Caspase-1. We observed a significant increase in ASC, Caspase-1, and Cleaved Caspase-1 levels compared to JEV-S1 (**Figure 2H**). Therefore, our data suggested that JEV-S1 and JEV-S3 differentially affect NLRP3 inflammasome activation in the brains of mice.

### Increased JEV-S3 replication is associated with increased infiltration of neutrophils in the spleen and brain

**Figure 3:**
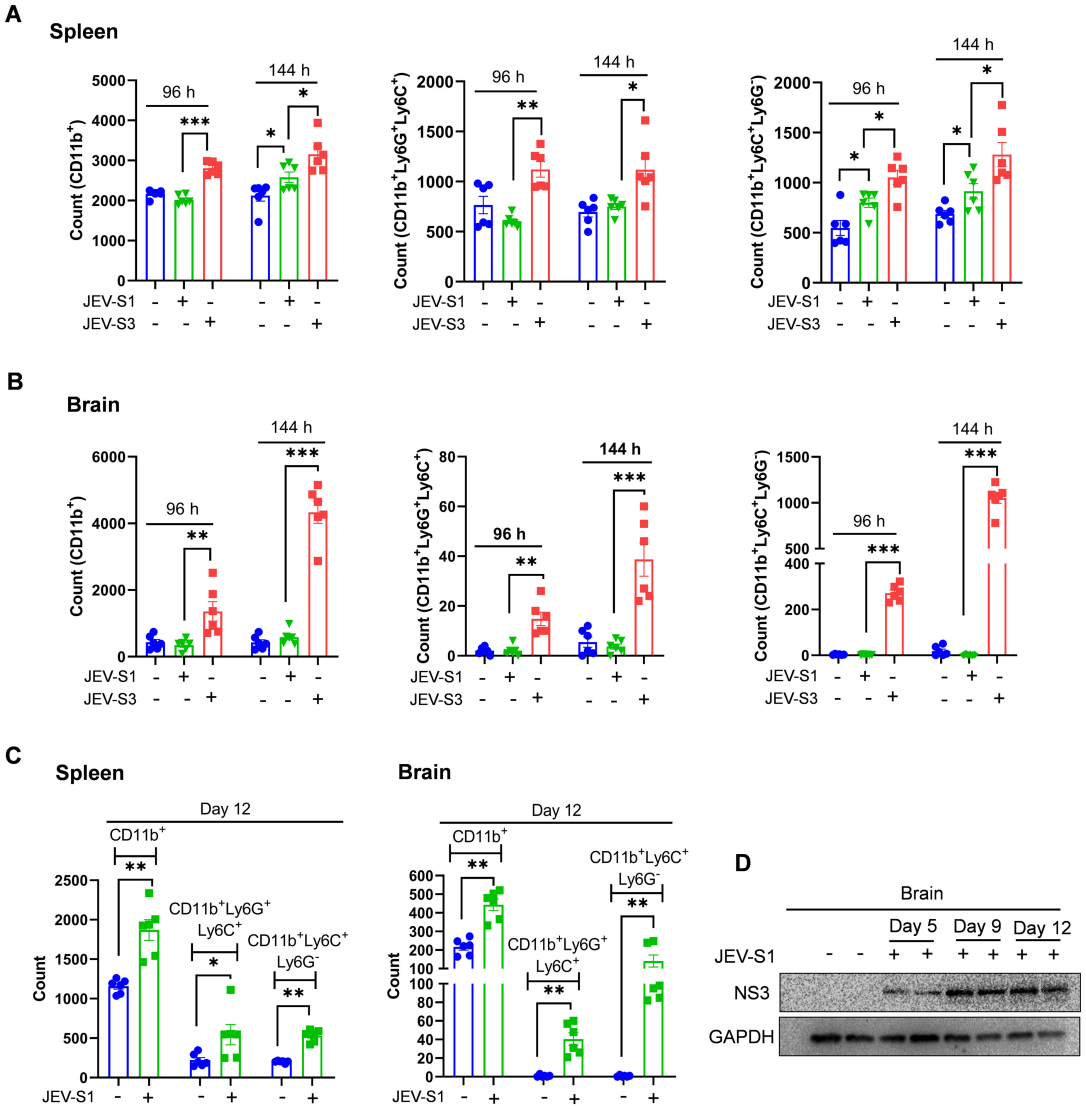
Increased JEV-S3 replication is associated with increased infiltration of neutrophils in the spleen and brain. Comparison of cell surface expression of markers CD11b, Ly6C, and Ly6G on spleen **(A)** and brain **(B)** cells from JEV-infected mice, harvested at 96 and 144 hpi. Bar graphs show the number of leukocytes expressing CD11b (left), Ly6G (middle), and Ly6C (right) in the spleen and brain. **(C)** Mice were infected with JEV-S1, and at day 12, the spleens and brains were harvested. The expression levels of CD11b, Ly6C, and Ly6G on spleen (upper panel) and brain (lower panel) were analyzed by FACS. Each dot in the bar graph represents cells from an individual mouse (n=6). Error bars are defined as SEM. P*-values* are calculated using an unpaired Student’s t-test; *P<0.05; **P<0.01, ***P<0.001. **(D)** Western blot showing levels of NS3 and GAPDH in JEV-S1-infected mice, harvested at different time points.

It was suggested earlier that viruses can enter the CNS by infecting and infiltrating peripheral immune cells (15). It could be possible that JEV-S1 and JEV-S3 infection cause significant differences in immune cell infiltration in the brain at early time points. To gain more insights into brain-infiltrating cells at an early time, we measured innate immune cells, monocytes, and neutrophils in the spleen and brain. The expression level of CD11b, neutrophils (CD11b^+^Ly6G^+^Ly6C^+^), and monocytes (CD11b^+^LyC^+^Ly6G^-^) was measured by FACS in spleen and brains at 96 and 144 hpi. We found an increase in the neutrophil (CD11b^+^Ly6G^+^Ly6C^+^) count in JEV-infected mice in the spleen and brain at later time points (**Figures 3A, B**, middle panel). Upon examination of the spleen for monocytes (CD11b^+^LyC^+^Ly6G^-^), the monocyte count increased in JEV-S1 and JEV-S3 at 96 hpi (**Figure 3A**, right panel), while the neutrophil (CD11b^+^Ly6G^+^Ly6C^+^) count increased at 144 hpi in JEV-S3 (**Figure 3A**, middle panel). We also found an increase in CD11b^+^, neutrophil (CD11b^+^Ly6G^+^Ly6C^+^) and monocytes (CD11b^+^Ly6C^+^Ly6G^-^) count in JEV-S3 at 96 and 144 hpi in the brain (**Figure 3B**). Thus, our results suggested that although JEV-S1 and JEV-S3 viral RNA and protein were detectable in different amounts in the brain at the same time point, JEV-S1 failed to attract neutrophils in the brain with significant numbers compared to JEV-S3. Interestingly, at day 12 post-infection, JEV-S1-infected mice became symptomatic and showed a substantial increase in CD11b, neutrophils and monocytes infiltration in the spleen and brain (**Figure 3C**). This is further supported by increased presence of viral protein in the JEV-S1-infected brain at day 12 post-infection (**Figure 3D**). Therefore, we conclude that JEV-S1 could be considered a less virulent strain than JEV-S3; however, under symptomatic conditions, both JEV-S1 and JEV-S3 can attract neutrophils and other immune cells from peripheral circulation to the brain.

### Neutrophil depletion accelerates encephalitis symptoms and death, irrespective of viral strains

**Figure 4:**
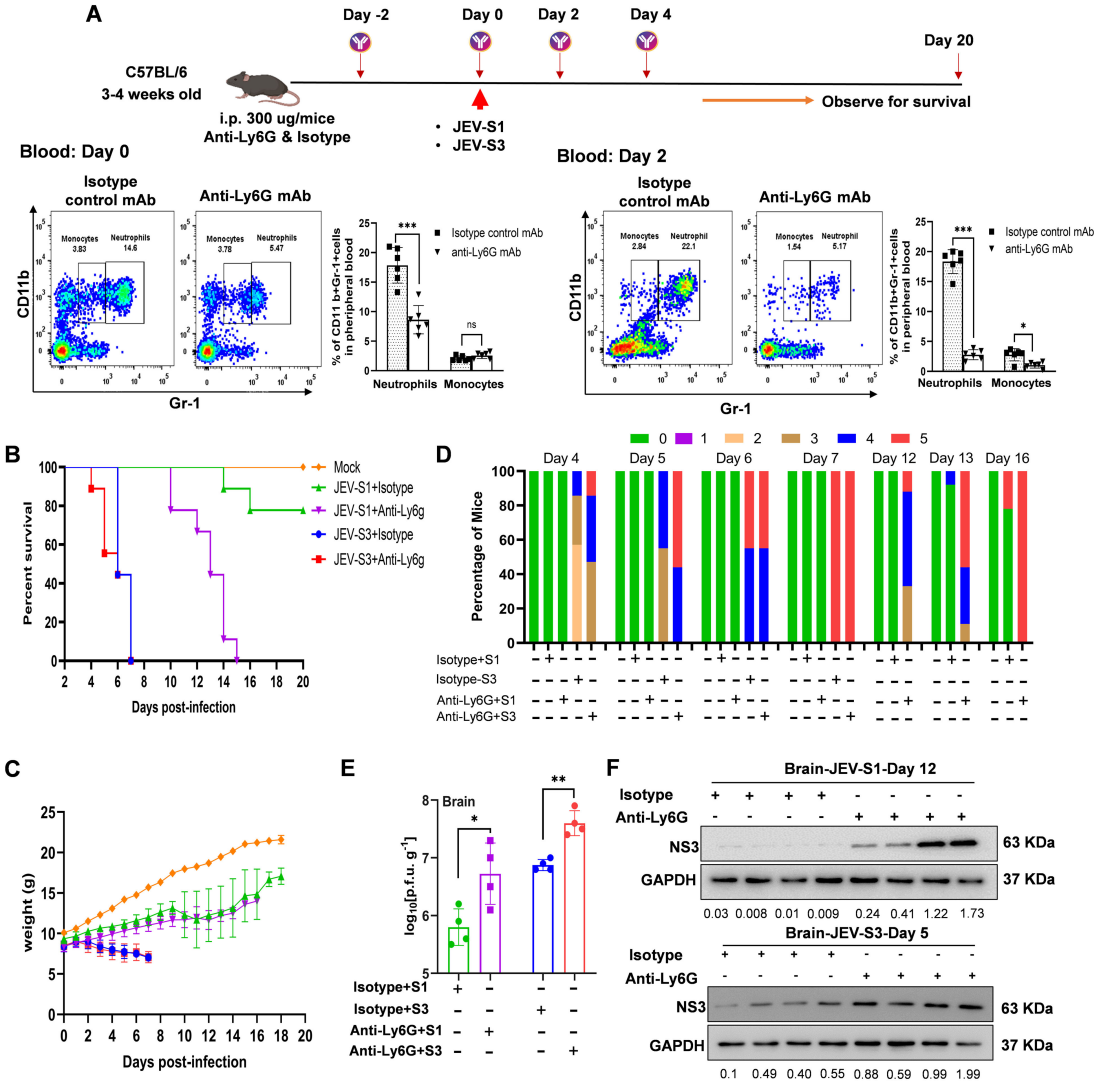
Neutrophil depletion accelerated encephalitis symptoms and death irrespective of viral isolates. **(A)** Schematic diagram representing experimental plans for neutrophil depletion in 3–4 week-old C57BL/6 mice. IP injection of Ly-6G and Isotype (300 μg/mouse) was given before 48 h of JEV infection. Then, on days 0 and 2, blood was drawn. Contour plots and Myeloid cell counts (bar graph) in peripheral blood in C57BL/6 (n=6 in each group) mice treated with either isotype control mAb or anti-Ly6G mAb by CD11b and anti-Gr1 at day 0 and day 2 were shown in the lower panel. P*-values* are calculated using an unpaired Student’s t-test; *P<0.05; **P<0.01, ***P<0.001. **(B)** Kaplan-Meier survival curve of Isotype and anti-Ly6G-JEV-S1 and JEV-S3 infected mice (n=9 in each group). **(C)** The change in the body weight of neutrophil-depleted JEV-S1 and JEV-S3 infected mice. **(D)** The Clinical Score is shown in neutrophil-depleted JEV-S1 and JEV-S3 infected mice. The color represents a score (0–5) related to the clinical symptoms; ‘0’ represents no symptoms, and 5 represents the most severe symptoms. **(E)** Virus yield was quantified by plaque assay. Virus titres in the infected brain tissues of mice are shown in a bar graph. Data in all bar graphs are presented as mean ± SD. P*-*values were calculated using two-tailed unpaired Student’s t-test; *P<0.05; **P<0.01; ***P<0.001. **(F)** The expression levels of NS3 and GAPDH were detected in the infected mice’s brain by immunoblotting. The corresponding molecular weight of the protein is shown on the right side.

Since neutrophils have been implicated in protective and pathological responses following virus infections (18), we investigated the role of neutrophils *in vivo* following infection with virus strains that differ in virulence in mice. Researchers have extensively used the mAb 1A8 clone (anti-Ly6G) to deplete neutrophils from mice. To compare the role of neutrophils following infection of mice with strains of different virulence, mice were treated with mAb 1A8 (anti-Ly6G) 48h before infection with 10^7^ pfu/ml of the virus. Briefly, mice were treated with mAb 1A8 (anti-Ly6G) (300 μ g/mice, ip route) two days before infection, and the antibody was given every second day post-infection. Control mice received an equivalent treatment with purified Isotype anti-rat IgG. Mice were monitored daily for signs of disease, changes in body weight, and survival (**Figure 4A**). Antibody treatment significantly reduced neutrophil levels in blood, marginally affecting monocyte levels (**Figure 4A**, lower panel). Depletion of neutrophils with 300 μg of anti-Ly6G was efficient in blood, with a reduction of ~14% & 22% (CD11b^+^Gr-1^hi^) in anti-rat IgG, while in mAb 1A8 (anti-Ly6G), it was ~5.47% & 5.17% (CD11b^+^Gr-1^hi^) at day 0 and 2, respectively. However, no significant changes in the number of monocytes (CD11b+Gr-1^int^) were observed at days 0 and 2 (**Figure 4A**, lower panel).

Mice infected with 10^7^ pfu/ml of JEV-S3 and treated with either mAb 1A8 or with control anti-rat IgG succumbed to infection 1 day earlier than anti-rat IgG-treated controls (**Figure 4B**). Mice infected with JEV-S3 and treated with either mAb 1A8 or anti-rat IgG lost weight rapidly (**Figure 4C**). All mice succumbed to the disease at day 7 post-infection; the clinical score is shown in **Figure 4D**. All mice infected with JEV-S1 treated with either mAb 1A8 or anti-rat IgG exhibited weight loss compared to mock mice. However, the weight loss and clinical manifestations seemed to be much less than the weight loss and clinical severity observed in mice infected with JEV-S3 (**Figures 4C, D**). Interestingly, all mice infected with JEV-S1 treated with mAb 1A8 died on day 15 post-infection, whereas only 30% died at 20 days post-infection (dpi) in the IgG-treated control animals (**Figures 4B–D**). These data indicate that Ly6G^high^ neutrophils play a beneficial role early in mice following challenges with JEV-S1 and JEV-S3 virus strains of moderate to high virulence.

To examine the effect of neutrophils on virus replication in the brain, we measured the level of infectious virus at the onset of severe symptoms (**Figures 4E, F**). Depletion of neutrophils allowed JEV replication to occur faster than in normal conditions. Interestingly, JEV-S1 in neutrophil-depleted mice exhibited increased viral replication, as evidenced by the increased viral titre and viral protein NS3 that were detectable in the brains (**Figures 4E, F**). Thus, our data suggest that neutrophils can be crucial in limiting viral replication in the brains of infected mice with JEV-S1 and JEV-S3. Depletion of neutrophils causes severe symptoms and accelerated death irrespective of viral isolates.

### Neutrophil depletion affects CD8 T-cell abundance in the brain of JEV-infected mice irrespective of viral isolates

**Figure 5:**
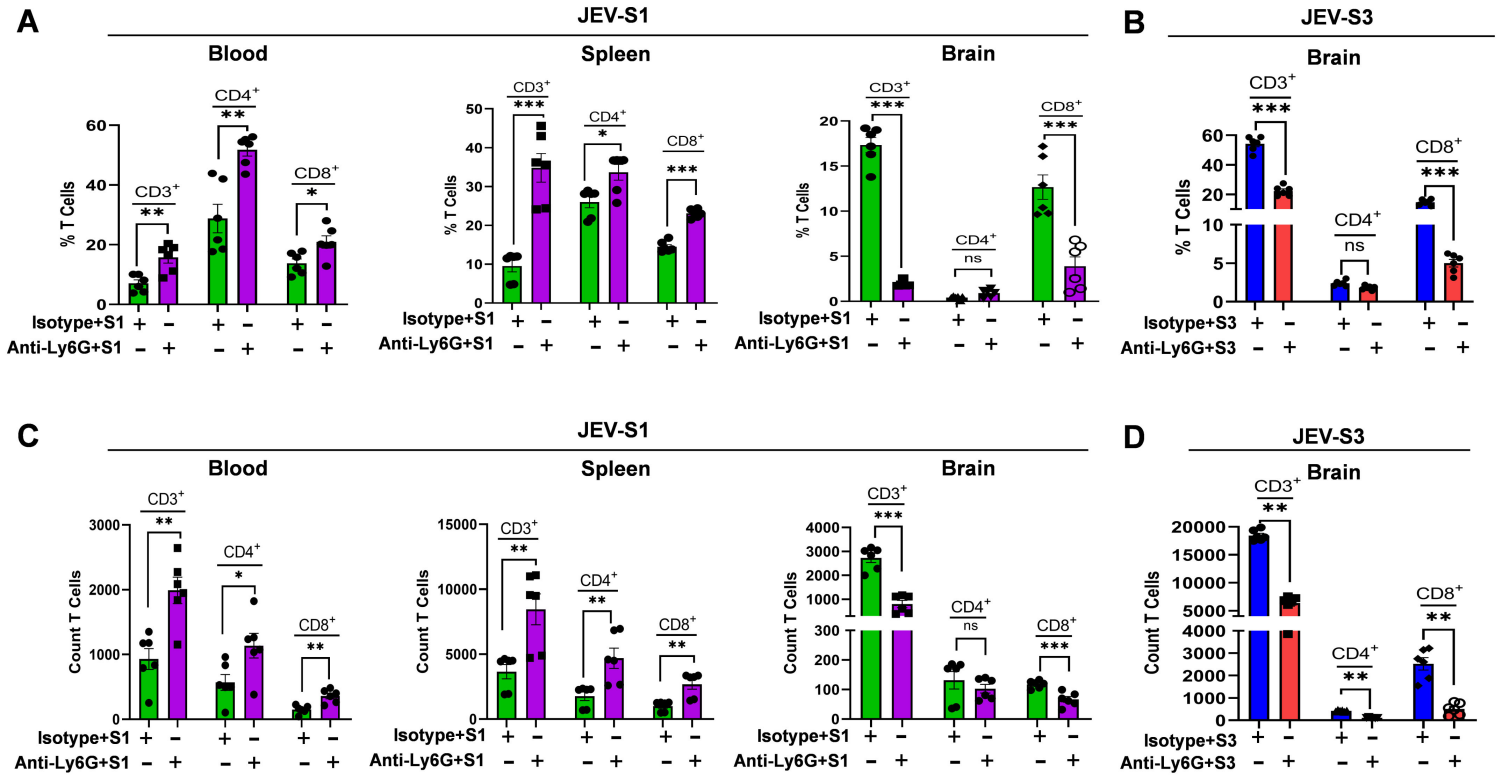
Neutrophil depletion affects CD8^+^ T-cell abundance in the brain of JEV-S3 and JEV-S1-infected mice. **(A)** Cell populations from the blood, spleen, and brain at the onset of symptoms in neutrophil-depleted JEV-S1 infected mice (n=6 mice in each group) were assessed by flow cytometry. CD11b, Ly6C, Ly6G, CD3, CD4, and CD8 T-cells were identified and described in the Materials and Methods section. **(B)** Cell population from the brain was assessed at the onset of symptoms in neutrophil-depleted JEV-S3-infected mice. The top **(A, B)** and bottom **(C, D)** panels represent % T cells and count for the same experiments. Error bars are defined as SEM. P*-values* are calculated using an unpaired Student’s t-test; *P<0.05; **P<0.01, ***P<0.001.

Neutrophil-mediated recruitment of T-cells into infected sites has been documented in bacterial and viral infections and chronic inflammatory diseases (19, 20). However, no such information is available on JEV infection. Neutrophils may provide chemotactic cues for efficient CD8^+^ T-cell migration (21). To understand if neutrophils could induce such an effect in JEV infection, we depleted neutrophils in 3–4 weeks of C57BL/6 mice. We investigated whether the absence of neutrophils resulted in equally impaired CNS-infiltrating immune cells. Therefore, compared to control animals treated with anti-rat-IgG, we characterized lymphocyte infiltration in the CNS of neutrophil-depleted mice during JEV-S1 and JEV-S3 infection. The absence of neutrophils in the CNS resulted in significant impairment of overall lymphocyte recruitment by reducing the total number of infiltrating CD3^+^ T-cells (~17.3% & ~56%) in rat-IgG; while in mAb A18, it was (~4.62% & ~23.3%) during JEV-S1 and JEV-S3 infection (**Figures 5A, B**). To detect a specific effect of neutrophil depletion on the inflammatory response in the CNS, we performed a detailed characterization of CNS-infiltrating lymphocyte subsets in anti-Ly6G and control-treated animals during sub-clinical and severe JEV infection. Of note, although there was a significant decrease in the number of leukocytes infiltrating into the CNS of anti-Ly6G-treated animals in comparison with IgG control-treated animals during JEV-S1 and JEV-S3 infection, we did not observe any difference in the frequencies of infiltrating CD4^+^ T-cells (**Figures 5A, B**). The absence of neutrophils in the CNS leads to a significantly reduced proportion of CD8^+^ T-cells (~12% & ~20% in rat-IgG vs ~2.82% & ~5% in mAb 1A8) in JEV-S1 and JEV-S3, respectively (**Figures 5B, D**). This indicates that neutrophil directly affects CD8 T-cell infiltration in the CNS. However, we did not observe a reduction in CD4 or CD8 levels in the blood and spleen (**Figure 5A**). CD3, CD4, and CD8 counts significantly increased in the blood and spleen (**Figure 5C**). The total number of infiltrating CD3^+^ T-cells in the spleen increased from ~8.41% to ~35.47% compared to anti-rat IgG during JEV-S1 infection. Also, the number of T-cell subsets, CD4^+^ T cells and CD8+ T cells, increased from ~25% to 32% and from ~14% to 24%, respectively. In blood, the CD3^+^ T-cells increased from 7.04% to ~15.19%, CD4^+^ was ~30% to ~50%, while CD8^+^ ~14.84% to ±20.93% in JEV-S1. This data suggested that, although neutrophil depletion increased CD4 and CD8 levels in the blood and peripheral organs, significant inhibition occurred in CD8+ T cell infiltration in the brain.

### Blocking the CXCR4 receptor with the specific inhibitor AMD3100 accelerates viral replication irrespective of the viral strain

**Figure 6:**
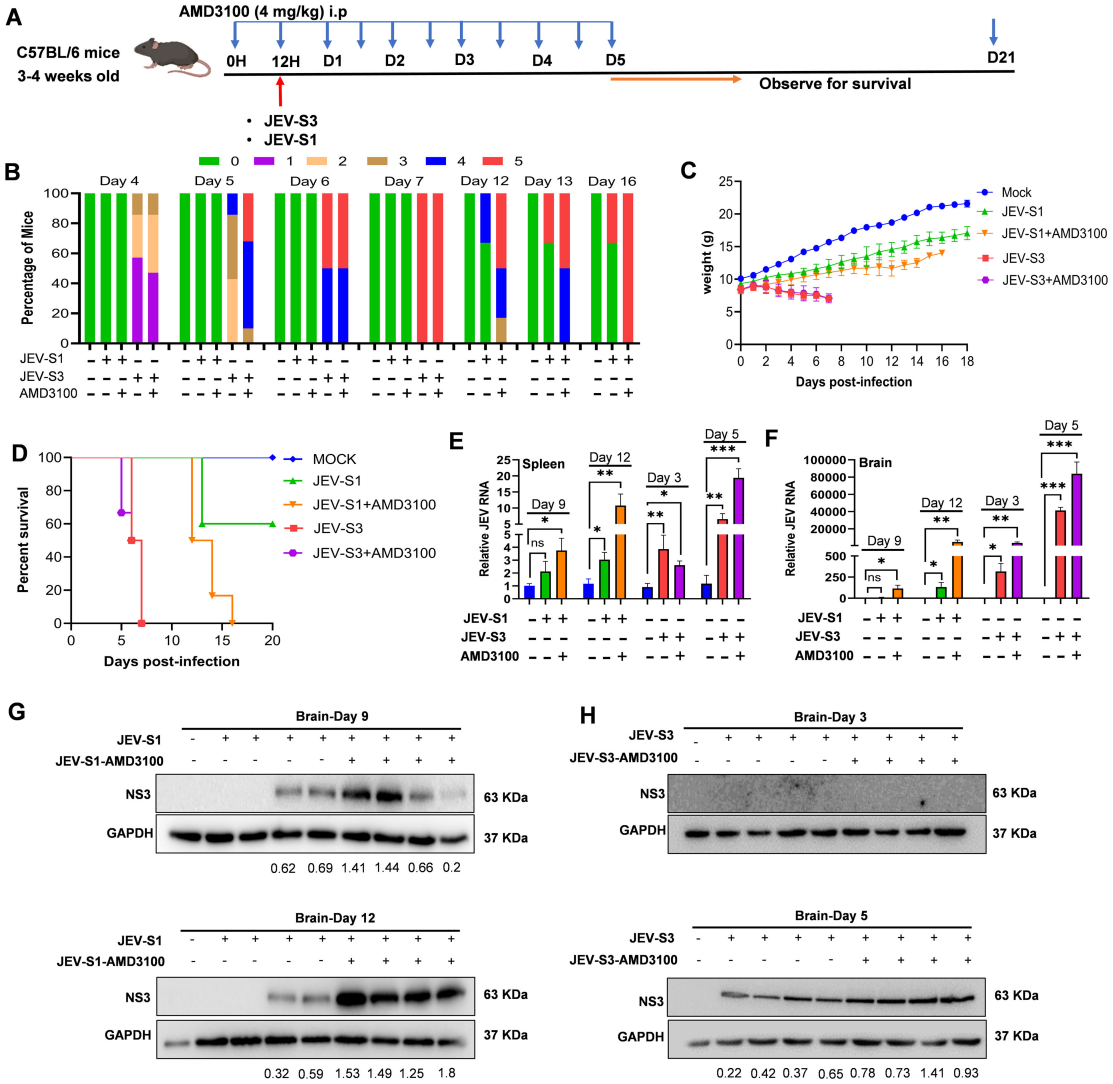
CXCR4 antagonist AMD3100 treatment increases JEV infection and decreases survival. **(A)** Schematic representation of experimental plans. C57BL/6 mice were treated intraperitoneally with AMD3100 at 4mg/Kg/mouse before 12 h of infection, and after every 12 h, AMD3100 was given till day 5. **(B)** The clinical score is shown in CXCR4 antagonist-treated JEV-S1 and JEV-S3-infected mice. The color represents a score (0–5) related to the clinical symptoms; ‘0’ represents no symptoms, and 5 represents the most severe symptoms. **(C)** The change in the body weight of JEV-S1 and JEV-S3-treated mice with CXCR4 antagonist mice. **(D)** Kaplan-Meier survival curve of CXCR4 antagonist-treated JEV-S1 and JEV-S3 infected mice (n=6 in each group). **(E, F)** The JEV mRNA was quantified by qRT-PCR in the spleen and brain at day 9 and day 12 post-antagonist-treated JEV-S1 and day 3 and day 5 in antagonist-treated JEV-S3-infected mice. The fold changes of JEV were normalized to the internal control of GAPDH. Data in all bar graphs are presented as mean ± SD. P*-*values were calculated using two-tailed unpaired Student’s t-test; *P<0.05; **P<0.01; ***P<0.001. **(G, H)** The expression levels of NS3 and GAPDH were detected in the infected mice’s brains by immunoblotting.

Significant inhibition of CD8^+^ T-cells in the brains of neutrophil-depleted mice may be linked to chemoattractant molecules such as CXCL12. CXCL12 binds with CXCR4 receptors and imparts the signal. It was previously reported that early recruitment of neutrophils into influenza-infected lungs is essential for CD8^+^ T-cell-mediated immune protection in mice because neutrophils make trails for CD8^+^ T-cells via CXCL12 (21). To further understand if the CXCL12-CXCR4 axis has any role in immune cell infiltration and viral pathogenesis, we used AMD3100, a specific inhibitor for CXCR4. We administered AMD3100 (4mg/kg) to mice through IP 12 hours before JEV infection. The inhibitor was given twice every day till day 5. Mice were monitored daily for signs of disease, changes in body weight, and survival (**Figure 6A**). Similar to neutrophil depletion, AMD3100 treatment caused severe symptoms. It reduced weight in mice irrespective of viral isolates (**Figures 6B, C**), and all the mice eventually died (**Figure 6D**). In a similar experiment, mice were harvested at different time points. Intracellular viral RNA was measured in the spleen and brain using RT-PCR (**Figures 6E, F**), and viral NS3 protein in brain lysates was checked by Western blot (**Figures 6G, H**). Compared to the untreated group, AMD3100-treated mice had increased levels of viral RNA and protein expression in the spleen and brains of both JEV-S1 and JEV-S3-infected mice (**Figures 6E–H**).

### CXCR4 antagonist AMD3100 treatment affects CD8 T-cell migration in the brain

**Figure 7:**
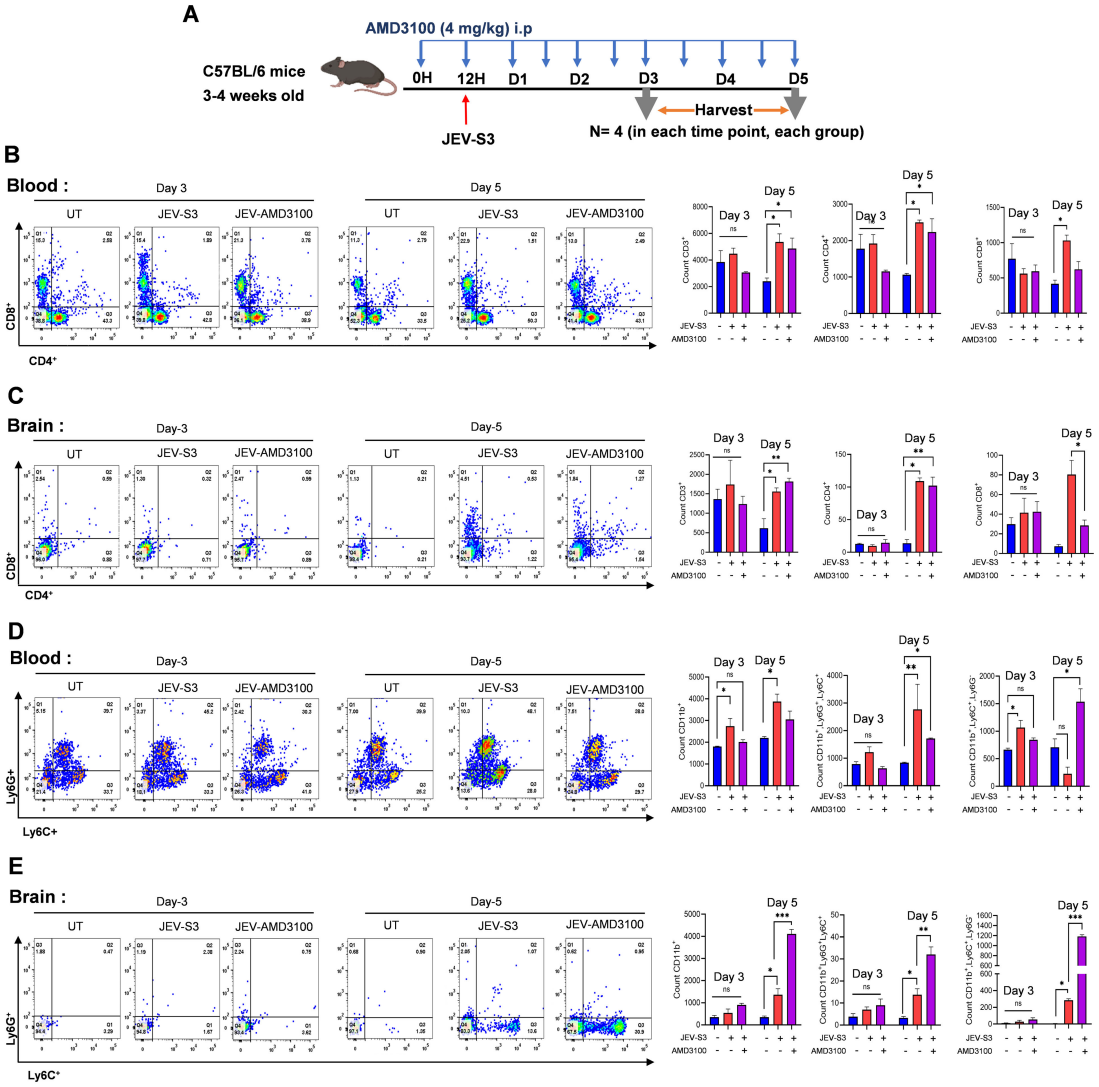
Flow cytometric analysis of immune infiltrating cells in JEV-S3-infected-treated brain. **(A)** Schematic diagram representing experimental plans. C57BL/6 (n=4 mice in each time-point) were treated intraperitoneally with AMD3100 at 4mg/Kg/mouse before 12 h of JEV-S3 infection. After every 12 h, the antagonist was given till day 5, and mice were harvested on days 3 and 5. **(B)** Comparison of cell surface markers CD11b, Ly6G, Ly6C, CD3, CD4, and CD8 on blood **(B, D)** and brain **(C, E)** cells from JEV-S3 and treated-AMD3100 infected mice. Data in all bar graphs are presented as SEM. Statistical significance was determined using a one-way ANOVA test: *P<0.05; **P<0.01; ***P<0.001.

Further, the effect of immune cell infiltration on AMD3100 treatment was studied in the infected mice treated with AMD3100. Mice were harvested at two different time points, and using FACS, we examined the immune cell status in the blood and brain. We chose two time points (for JEV-S3 days 3 and 5) based on the appearance of mild and severe symptoms in infected mice (**Figure 7A**). As shown in **Figure 7B**, CD4^+^ and CD8^+^ T cell levels did not change significantly in blood and brain at day 3 post-infection in mock, JEV-S3-infected, treated, or untreated groups. In contrast, increased CD4^+^ level was observed in the blood and brain of JEV-S3-infected mice on day 5 post-infection. Interestingly, though an elevated level of CD8^+^ T cells was observed in blood in JEV-S3-infected mice in both treated and untreated groups compared to mock, CD8^+^ T cell levels in the brain were significantly reduced in AMD3100-treated mice (**Figure 7C**). We also checked the myeloid population in blood and brain of treated and untreated mice. Although the CD11b^+^ population increased in the blood of infected mice on day 3 and day 5 post-infection, the number of CD11b^+^ cells increased on day 5 in the infected mice’s brains, indicating peripheral immune cell infiltration in the brain. The CD11b^+^ population was higher in AMD3100-treated mice in infected brains than in untreated mice. Among the CD11b^+^ population, we further gated based on the Ly6G^+^ and Ly6C^+^ populations. We did not find much difference in the neutrophil population in the blood and brain of treated and untreated mice at day 5 post-infection. However, increased monocyte accumulation (CD11b+Ly6C^+^Ly6G^-^) was noted in AMD3100-treated mice brains (**Figures 7D, E**).

**Figure 8:**
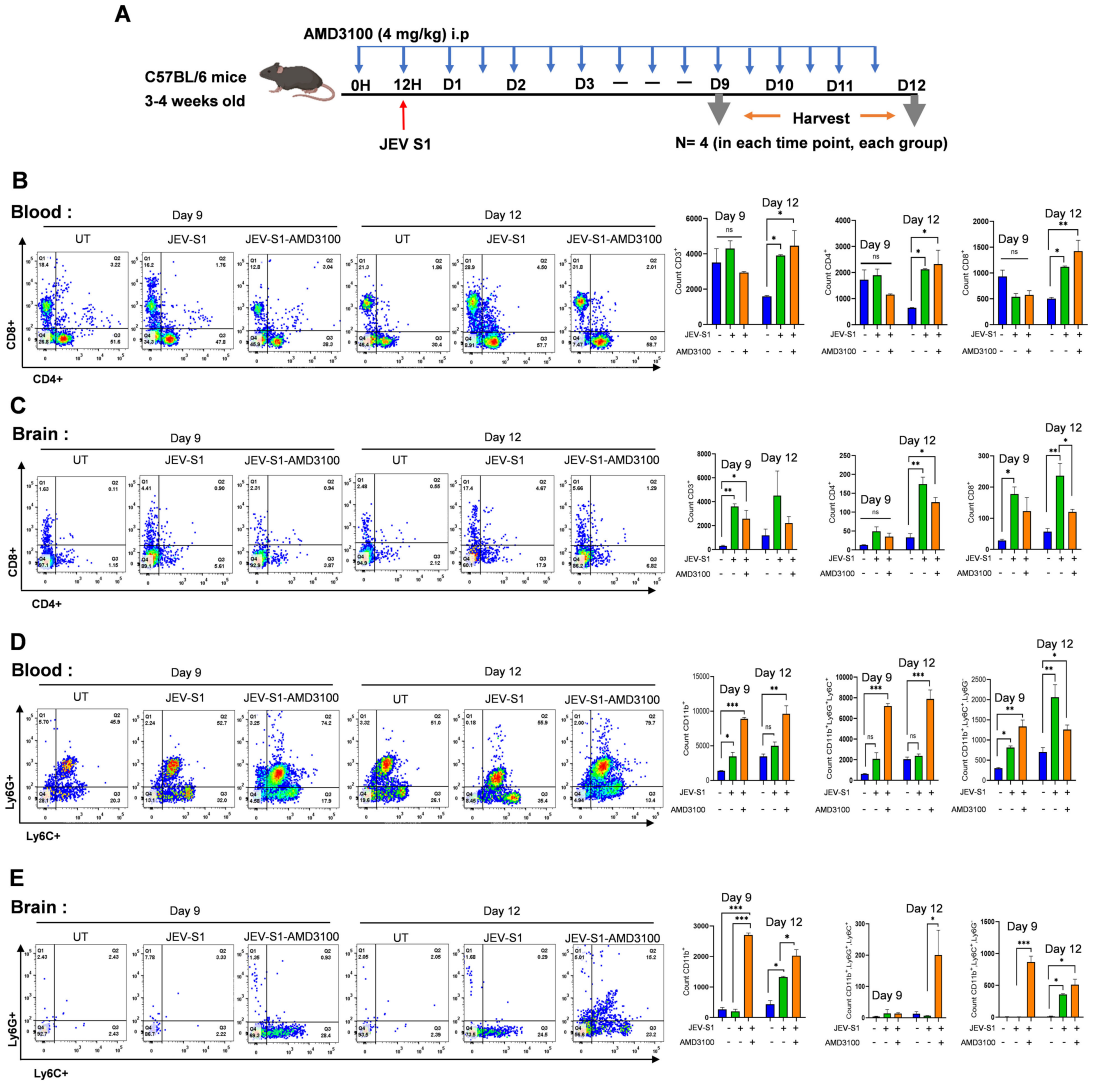
Immune cell abundance and infiltration in blood and brain, respectively, in JEV-S1-infected-treated mice. **(A)** Schematic diagram representing experimental plans. C57BL/6 (n=4 mice in each time-point) were treated intraperitoneally with AMD3100 at 4mg/Kg/mouse before 12 h of JEV-S1 infection, and after every 12 h, the antagonist was given till day 11. Mice were harvested on days 9 and 12. **(B)** Quantification of cell surface markers CD11b, Ly6G, Ly6C, CD3, CD4, and CD8 on blood **(B, D)** and brain **(C, E)** cells from JEV-S1 and treated-AMD3100 infected mice. All data are expressed as SEM; statistical significance was determined using a one-way ANOVA test: *P<0.05; **P<0.01; ***P<0.001.

In another experiment, mice were infected with JEV-S1 and given treatment with AMD3100, as described in the Materials and Methods section. Mice were harvested on day 9 and day 12, based on the appearance of mild and severe symptoms in infected mice (**Figure 8A**). Immune cell infiltration was studied using FACS. Similar to JEV-S3-infected mice, no change in CD4^+^ and CD8^+^ levels was observed in the blood and brain of treated and untreated groups of JEV-S1-infected mice. However, a decreasing trend in CD8^+^ T cell level was noted in the brains of AMD3100-treated mice at 12 dpi (**Figures 8B, C**). Compared to JEV-S3 mice, both neutrophil and monocyte populations were elevated in the blood and brains of AMD3100-treated JEV-S1-infected mice (**Figures 8D, E**).

### Neutrophil depletion or treatment with a CXCR4 antagonist alters NLRP3-mediated inflammation in the brai

Since neutrophil depletion or AMD3100 treatment augmented viral replication and symptoms (**Figures 6G, H**), we further checked if neutrophil depletion or AMD3100 treatment impacts neuroinflammation. In infected mice, NLRP3 inflammasome formation contributed significantly to JEV pathogenesis. We administered C57BL/6 mice with an isotype control antibody or anti-Ly-6G (1A8) antibody to deplete neutrophils specifically. In a separate experiment, mice were treated with AMD3100 for 12 hours before JEV infection. In both cases, mice were challenged with JEV-S1 and JEV-S3 (10^7^ pfu/ml) through the intraperitoneal route. Mice’s brains were harvested, and NLRP3 inflammasome gene levels were quantified.

**Figure 9:**
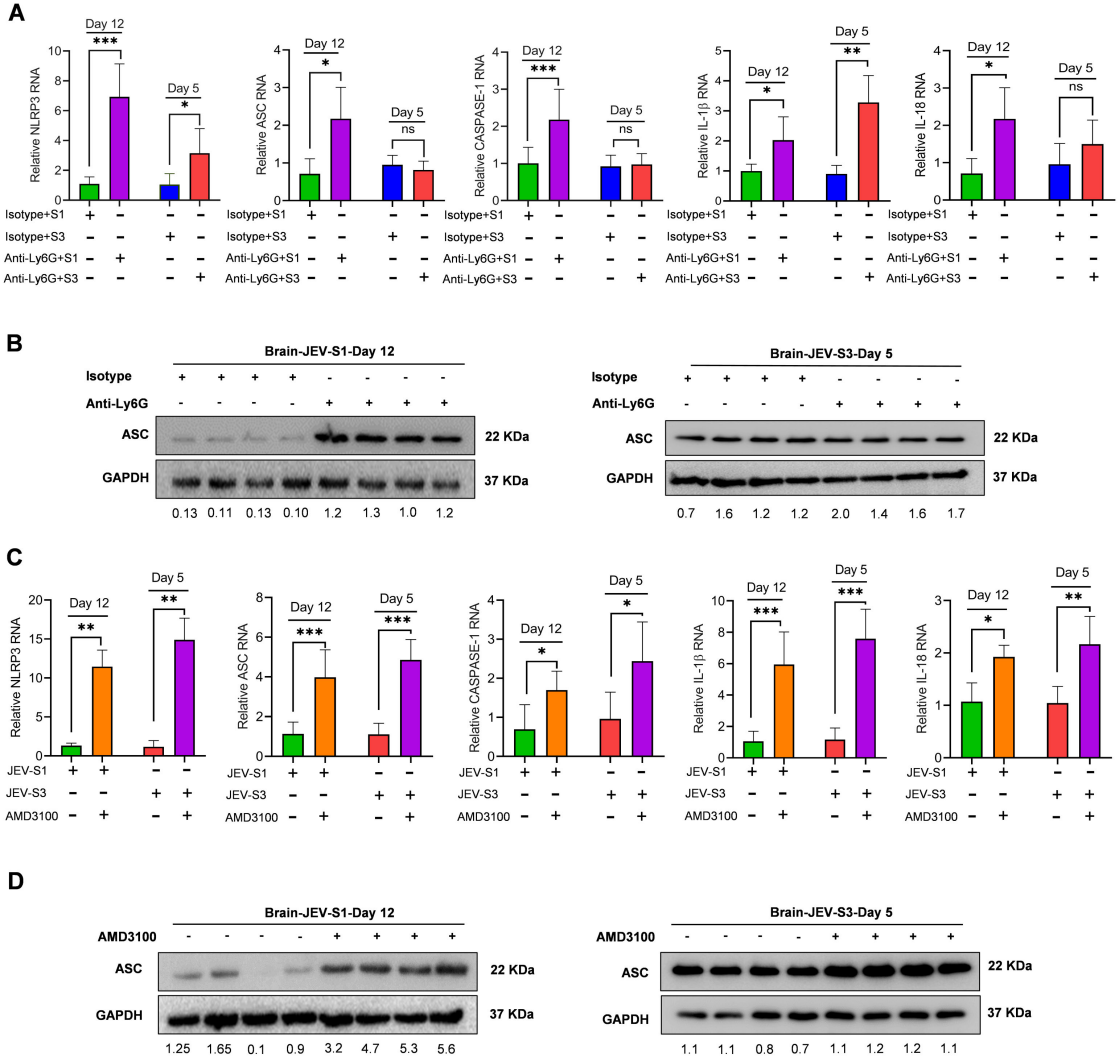
Induction of neutropenia or treatment with CXCR4 antagonist augmented NLRP3-mediated inflammation in the brain. **(A)** The NLRP3 inflammasome genes were quantified using qRT-PCR in the brain in Isotype and neutrophil-depleted JEV-S1 and JEV-S3 infected mice (n=4 in each group) at day 12 and day 5, respectively. **(B)** The expression levels of ASC and GAPDH were detected in infected mice brain by immunoblotting. **(C)** NLRP3 inflammasome genes were quantified by qRT-PCR in JEV-S1, JEV-S3, and AMD3100-infected mouse brains. P*-*values were calculated using two-tailed unpaired Student’s t-test; *P<0.05; **P<0.01; ***P<0.001. **(D)** The ASC and GAPDH expression levels were detected in infected mice brain by immunoblotting.

There was a significant increase in NLRP3 and IL-1β by 3-fold each in mAb 1A8 JEV-S3-infected mice. Similarly, there was a substantial increase in NLRP3 transcript by 7-fold while ASC, Caspase-1, IL-18, and IL-1β were increased by 2-fold each in mAb 1A8 JEV-S1 infected mice, suggesting a robust inflammation upon neutrophil depletion in JEV-S1 infected mice’s brain (**Figure 9A**). However, we did not observe an increase in ASC, Caspase-1, and IL-18 at transcript levels. To estimate the ASC protein level, we carried out an immunoblot of total protein isolated from mice brains at the onset of symptoms. In JEV-S3, there was no significant change in mAb 1A8 compared to anti-rat IgG. Still, there is a substantial increase in ASC level in mAb 1A8 compared to anti-rat IgG during JEV-S1 infection (**Figure 9B**).

There was a significant increase in transcript level of NLRP3, ASC, Caspase-1, IL-18, and IL-1β in AMD3100-treated JEV-S1 and JEV-S3-infected mice brain (**Figure 9C**). Also, significant changes in ASC at the protein level are evident (**Figure 9D**). Our data confirmed that neutrophil depletion or treatment with the CXCR4 inhibitor significantly augmented NLRP3 inflammation in the brain irrespective of viral strain.

### Chemokine levels increased in JEV-infected mice but failed to show a reduction in neutrophil-depleted or AMD3100-treated mice

**Figure 10:**
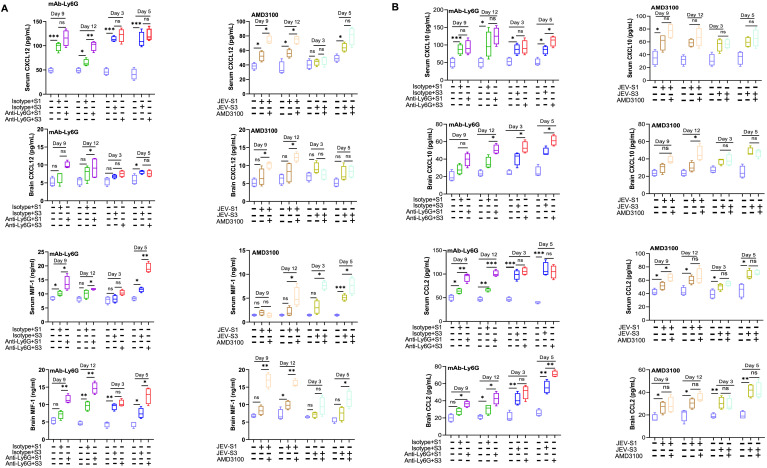
Chemokine levels in serum and brain lysates. **(A)** ELISA measured the concentration of CXCL12 (Top) and MIF-1 (bottom) levels in neutrophil-depleted mice, and AMD3100-treated infected mice serum (upper panel) and brain lysate (lower panel) plotted as a box plot. The concentration of MIF-1 level was measured using ELISA in neutrophil-depleted and AMD3100-treated infected mice serum (upper panel) and brain lysate (lower panel). **(B)** CXCL10 and CCL2 levels in serum (upper panel) and brain lysate (lower panel) in neutrophil-depleted mice and AMD3100-treated infected mice were measured by ELISA. The concentration of CXCL10 and CCL2 was plotted as a box plot. Each group has serum and brain lysate from (n=4) different infected mice. P*-*values were calculated using two-tailed unpaired Student’s t-test; *P<0.05; **P<0.01; ***P<0.001.

During viral infection, the chemokine gradient may guide immune cells to the site of infection. To identify if specific chemokines have any role in immune cell migration into the infected mice’s brain, we measured the levels of different chemokine factors, including CXCL12 and macrophage migration inhibitory factor-1 (MIF-1), in infected mice serum and brain lysates (**Figure 10A**). MIF induces chemotactic responses, promoting the recruitment and migration of immune cells, including monocytes and neutrophils, to inflammatory sites (22). MIF can act as a non-cognate ligand for CXCR4, influencing the targeted migration of T cells via CXCR4 and, thus, being involved in the adaptive immune response to infections. On the other hand, CXCL12 specifically binds to the CXCR4 receptor, and this engagement facilitates the migration of immune cells (23, 24). Compared to mock, severe JEV infection (day 12 for JEV-S1 and day 5 for JEV-S3) increased CXCL12 levels in serum. With neutrophil depletion or AMD3100 treatment, we observed either an increase (in the case of JEV-S1) or no change (in the case of JEV-S3) in CXCL12 in serum and brain, suggesting that other infiltrating immune cells might have an impact on CXCL12 levels.

On the other hand, with neutrophil depletion or AMD3100 treatment, MIF-1 level was significantly induced in serum and brains of infected mice irrespective of viral strains (**Figure 10A**, left panels). Klein et al. reported that neurons can secrete CXCL10 in response to WNV infection, which helps in trafficking CD8+T cells to the brain and helps in clearing WNV (25). Another chemokine, CCL2, can be induced during neuroinflammation to recruit immune cells into the brain (26). Since we did not observe a reduction in CXCL12 or MIF-1 levels upon neutrophil depletion, we checked two additional chemokines, CXCL10 and CXCL2, in the brain. However, similar to CXCL12 and MIF-1, we did not observe a reduction in levels in serum and brain upon neutrophil depletion or AMD3100 treatment (**Figure 10B**). However, both chemokines were significantly induced in serum and brain upon JEV infection, irrespective of viral strains. Several reports suggested that, other than neutrophils, endothelial cells, monocytes, and brain cells (25, 27, 28) can produce chemokines, including MIF-1, CXCL10, CCL2, and CXCL12. Thus, neutrophil depletion did not impact chemokine abundance in the brain, irrespective of viral strains.

### Brain-infiltrating CD8^+^ T-cells decrease the expression of CXCR4 during neutrophil depletion

**Figure 11:**
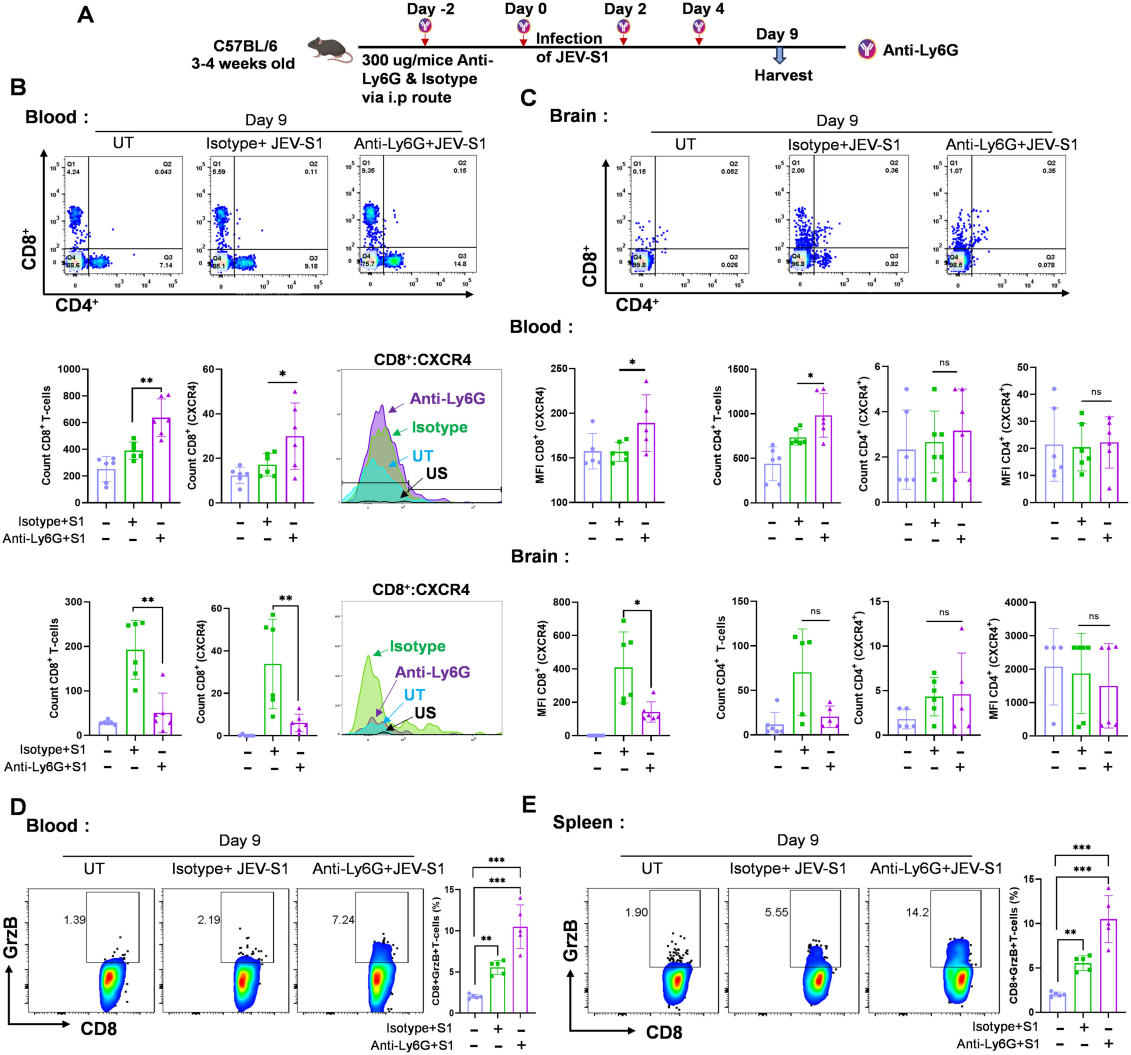
CXCR4 expression pattern in neutrophil-depleted mice in blood and brain. **(A)** Schematic representation of experimental plans: IP injection of anti-Ly6G and Isotype 300 µg/mice (n=6 in each group) were given before 48 h of JEV-S1 infection, and antibodies were given as depicted in the schematic figure. **(B, C)** The contour plots represent the percentage of CD4+ and CD8+T cells at day 9 from blood and brain (upper panel). The count of CXCR4^+^ cells was determined in CD8^+^ and CD4^+^ T cells; quantification of CXCR4^+^ cells on CD8^+^ and CD4^+^ T cells in blood and brain is shown in the lower panel. **(D, E)** Intracellular and surface staining of CD8 T-cells from Blood and spleen for Granzyme B, at day 9 post-infection, respectively. P*-*values were calculated using two-tailed unpaired Student’s t-test; *P<0.05; **P<0.01; ***P<0.001.

CXCL12 has potent chemoattractant properties for cells expressing CXCR4, such as monocytes and lymphocytes. Since we did not observe a decrease in CXCL12 or MIF level, a ligand for CXCR4, we looked into CXCR4 levels in infiltrating immune cells. We depleted neutrophils at the early stages of JEV-S1 infection, as shown in **Figure 11A**, and levels of CXCR4 in CD4^+^ and CD8^+^ T-cells were investigated. Upon neutrophil depletion, CD4^+^ and CD8^+^ counts went up significantly in the blood. However, to our surprise, CXCR4 levels were reduced considerably in CD8^+^ T cells compared to CD4^+^ T cells (**Figure 11B**, top and bottom panels).

On the other hand, neutrophil depletion significantly reduced CD8^+^ numbers and CXCR4 levels in the brain (**Figure 11C**). We also checked CXCR4 expression on monocytes of JEV-S1-infected mice. We observed increased monocyte levels in the blood and more infiltration in the brain. Increased monocyte infiltration was also correlated with increased CXCR4 expression in the monocytes (Data not shown). Thus, our data confirms that a lower CD8^+^ count in infected brains of neutrophil-depleted mice may be due to reduced CXCR4 expression on CD8^+^ cells and not CXCL12. Next, we looked into the mechanisms of the reduction of CXCR4 levels in CD8+ T cells. We found in the literature that down-regulation of CXCR4 expression may be possible when the cells undergo peripheral differentiation, and low CXCR4 expression leads to effector CD8 T cells (29). We checked the Granzyme B (GrB) level in CD8, as when the CD8* cells become activated, they release more GrB to kill the infected cells. We performed intracellular staining of cells in the blood and spleen (**Figures 11D, E**). As compared to mock, IgG + JEV-infected mice exhibited an increased level of GrB in the blood and spleen. However, in neutrophil-depleted mice, GrB level augmented 3-fold as compared to the IgG + JEV infected group, suggesting that in the absence of neutrophil, CD8 cells become more activated and during this process they lose CXCR4 marker expression.

### Contribution of CD8+ T-cells to the control of JEV infection

**Figure 12:**
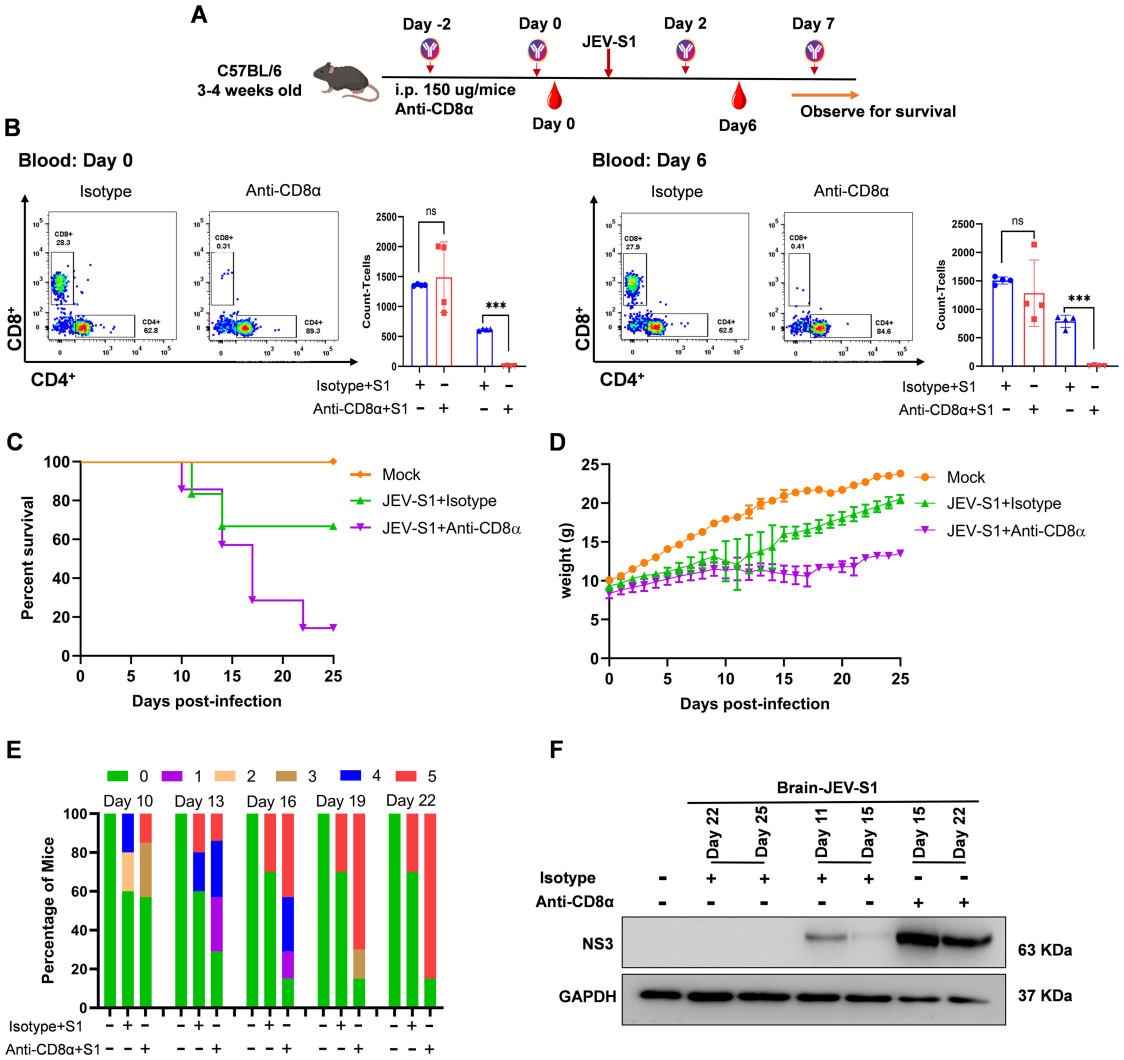
Contribution of CD8^+^ T-cells to control of JEV infection. **(A)** Schematic representation of experimental plans for CD8 depletion. IP injection of anti-mouse CD8α and Isotype (150 µg/mouse) was given before 48 h of JEV-S1 infection, and antibodies were given as depicted in the schematic figure. **(B)** Contour plots represent the percentage of CD4 and CD8+ cells. Lymphocyte cell counts (n=4 in each group) in peripheral blood at day 0 and day 6, depicted as a bar graph. P*-values* are calculated using an unpaired Student’s t-test; ***P<0.001. **(C)** Kaplan-Meier survival curve of isotype and anti-CD8α-JEV-S1 infected mice (n=7 in each group). **(D)** The change in the body weight of CD8^+^ T-cells depleted of JEV-S1 and isotype-infected mice. **(E)** The Clinical Score is shown in the CD8^+^ T-cells-depleted group and isotype-infected mice. The color represents a score (0–5) related to the clinical symptoms; ‘0’ represents no symptoms, and 5 represents the most severe symptoms. **(F)** The expression levels of NS3 and GAPDH were detected in the infected mice brains by immunoblotting.

Since the CD8 cell number was reduced in JEV-infected brains upon neutrophil depletion, we also looked into the contribution of CD8 to JEV pathology. To establish the role of CD8^+^ T-cells in the later phase of JEV infection, C57BL/6 mice were treated with anti-mouse CD8α (150 μg/mice) 48 h before the infection with 10^7^ pfu/ml of JEV-S1; the antibody was given, as depicted in **Figure 12A**. Control mice received an equivalent treatment with purified anti-rat IgG. Mice were monitored daily for signs of disease, changes in body weight, and survival. Antibody treatment significantly reduced CD8^+^ T-cell levels in blood without affecting CD4^+^ T-cell levels (**Figure 12B**). Depletion of CD8^+^ T-cells with 150 μg of anti-mouse CD8α was efficient in blood, with a reduction of ~28.3 & 27.9% (CD8^+^) in anti-rat IgG, while in anti-mouse CD8α, it was ~0.31 & 0.41% (CD8^+^) at day 0 and 6, respectively. There is a decrease in CD3^+^ T-cell levels in the depleted group. However, no significant changes in the level of CD4^+^ T-cells on days 0 and 6 were observed (**Figure 12B**). Thus, when CD8^+^ T-cells were depleted *in vivo*, they were challenged with 10^7^ pfu/ml of JEV-S1. We have observed a significant increase in susceptibility to JEV-S1 infection in mice compared to mice treated with a JEV-S1+Isotype control antibody (90% and 30% mortality rates) (**Figure 12C**). As shown in **Figure 12D**, all the mice in a depleted group show a significant decrease in body weight from day 10 compared to the isotype control. Mice in the CD8^+^ depleted group show higher clinical scores than infected isotype control (**Figure 12E**). Thus, CD8^+^ T-cells demonstrate a beneficial role in later stages of infection. Reduction in CD8 levels increases the virulence of the JEV-S1 strains, and mice eventually die due to increased viral load in the brain (**Figure 12F**).

## Discussion

An interaction between the pathogen and the host immune cells dictates the outcome of the viral infection. In this study, we examined the dynamics of human neutrophil and their impact on peripheral immune cell infiltration during mild and severe JEV. Using two well-characterized mice-adapted JEV isolates (JEV-S1 & JEV-S3), mimicking subclinical and severe infection, we demonstrated that neutrophil infiltration in different organs correlates with increased viral replication. An increase in neutrophil infiltration in the spleen and brain was observed later in mice infected with JEV-S3, causing severe symptoms. On the other hand, the increased neutrophil level was noted in the spleen of JEV-S1-infected mice at day 12 post-infection, and less neutrophil infiltration was observed in the brain compared to mice infected with JEV-S3. Both the protective and detrimental roles of neutrophils are reported in the literature. The protective effect of neutrophils was demonstrated in the study using AG129 interferon/receptor-deficient mice infected with the ZIKA strain. Results from this study suggest that neutrophils play a role in mitigating or protecting ZIKV-infected AG129 mice from motor deficits, probably by enhancing immune responses that reduce viral load (30). Studies using the WNV virus show that neutrophils will respond biphasically during infection. Neutrophils can serve as a reservoir for replication and dissemination in early infection and later contribute to viral clearance (9). Enhanced protection from WNV encephalitis could be achieved by limiting neutrophil trafficking to the CNS.

Infection of neutropenic animals with JHMV resulted in increased virus replication and mortality compared with control mice, indicating that neutrophils are crucial for limiting virus replication (12).

The increase in immune cell infiltration is likely due to a sharp rise in neutrophil chemoattractant production. Neutrophils promote effector CD8+ T cell migration into the influenza-infected trachea (21)by releasing chemokine trails. Also, neutrophils can play differing roles in modulating influenza disease depending on the virulence of the particular strain examined. Influenza virus strains of low or high virulence in mice differed markedly in their ability to recruit neutrophils to the airways (31).

Considering the critical roles of neutrophils in disease outcomes, the current study contributes several essential insights to our understanding regarding the role of neutrophils during JEV infection of mice. We have compared outcomes following the treatment of mAb 1A8 (anti-Ly6G, specific for neutrophils) and report that depletion of Ly6G^+^ cells in mice can be associated with increased virus replication and poor outcomes. Neutrophils are known to affect immune cell infiltration at the site of infection by paving the chemokine gradient. Our study also highlighted that neutrophil depletion affects CD8 cell abundance in infected mice and reduces CXCR4 expression on CD8 T cells. Although we did not observe a reduction in CXCL12, a chemokine that acts as a ligand for CXCR4, is critical for immune cell migration (32, 33). CXCL12 and CXCR4 expression regulation in cerebral endothelial cells plays a vital role in CD8 migration across the blood-brain barrier (33). CXCL12 stimulated the transmigration of monocytes and lymphocytes (34). In our study, CLCX12 serum and brain levels augment in neutrophil-depleted mice infected with JEV-S1 on day 12, but no changes are observed in JEV-S3-depleted mice on days 3 or 5. However, the different impact of the virus strain on endothelial cells could be one of the possible reasons. Silwedel et al. (35) also observed that LPS of E. coli O55:B5 caused a decreased level of CXCL12 after 4 hours’ treatment, which resulted from the increase of leukocyte migration, suggesting that leakiness in brain microvascular endothelial cells reduced CXCL12 expression.

As JEV-S3 is a more virulent strain, it showed BBB breaching within 4–5 days post-infection (**Figure 2**), and mice died within 24h post-development of symptoms. Neutrophil depletion enhances viral load in the brain, and severity increased significantly (**Figure 4**). JEV-S3 significantly reduced the tight junction protein on endothelial cells. In contrast, JEV-S1 has a minimal effect. This difference may contribute to increased CXCL12 levels in JEV-S1-infected mice compared to JEV-S3 infection.

Reduced CD8 levels in neutrophil-depleted mice brains may result from reduced CXCR4 expression on CD8 cells. Studies have shown that CD8 T cells play a protective role during primary JEV infection, possibly by preventing the breach in the BBB and neuronal damage (36). We found that neutrophil depletion augmented GrB level in CD8 cells, indicating they are differentiating into effector cells. During this differentiation process, they probably lose CXCR4 expression (29). Reduced CXCR4 expression allows less CD8 migration into the brain. We observed that neutrophil depletion affects the abundance of CD8 cells in the infected brain. Therefore, to understand the contribution of CD8 in JEV outcomes, we depleted CD8 using CD8-specific antibodies. We observed that CD8 depletion does not accelerate the development of severe symptoms in mice infected with JEV-S1.

However, approximately 90% of mice in the CD8-depleted group eventually died, while 20% died in the wild-type mice. Also, compared to neutrophil-depleted mice (**Figure 4**), CD8-depleted mice showed a significant delay in death (day 15 vs day 25). This suggests that neutrophil depletion, combined with reduced CD8 migration in the brain, enables the virus to replicate more efficiently and rapidly than in the group with only CD8 depletion. This is likely due to the limited early and late immune responses, resulting in a faster death rate in the neutrophil-depleted group. CD8 is perhaps involved in the later stage of viral infection when the virus is detectable in the brain. As neutrophils reach the brain, they attract more CD8 cells to clear the virus from the infected brain. In the absence of CD8 T cells, we also observed an increase in viral load, unlike in the presence of CD8 T cells (**Figure 12F**), which may result in a worsening of the condition and ultimately lead to death. Further study is needed to clarify the virulence factors that influence CXCR4 expression in CD8 cells. It would be interesting to investigate whether the adoptive transfer of CD8^+^ T cells from CXCR4 knockout donor mice into TCRβ-null recipient mice enhances viral replication.

Our study also does not clarify whether CXCR4^low^ CD8 cells are phenotypically and functionally different from those in normal conditions. A recent study suggested that myeloid-derived suppressor cells (MDSCs) can modulate CD8 phenotypes and function in the JEV infection model (37). Previous studies also indicated that the outcome of JEV infection, death or survival, was determined by qualitative differences in CDR3 amino acid sequences of brain-infiltrating T-cells (38). Therefore, it will be interesting to study the TCR repertoire and T-cell clone frequency between mice that showed subclinical and severe symptoms in the future. CD8+ T cells play a vital role in JEV infection. A multicentric study demonstrated that JEV-induced encephalitis presents a deficiency in CD8+ T cell response, whereas individuals’ CD4^+^ T cell response is associated with good recovery from JE in humans. These differences strengthen the importance of CD8+ T cells in the immunological response to JEV (39).

**Figure 13:**
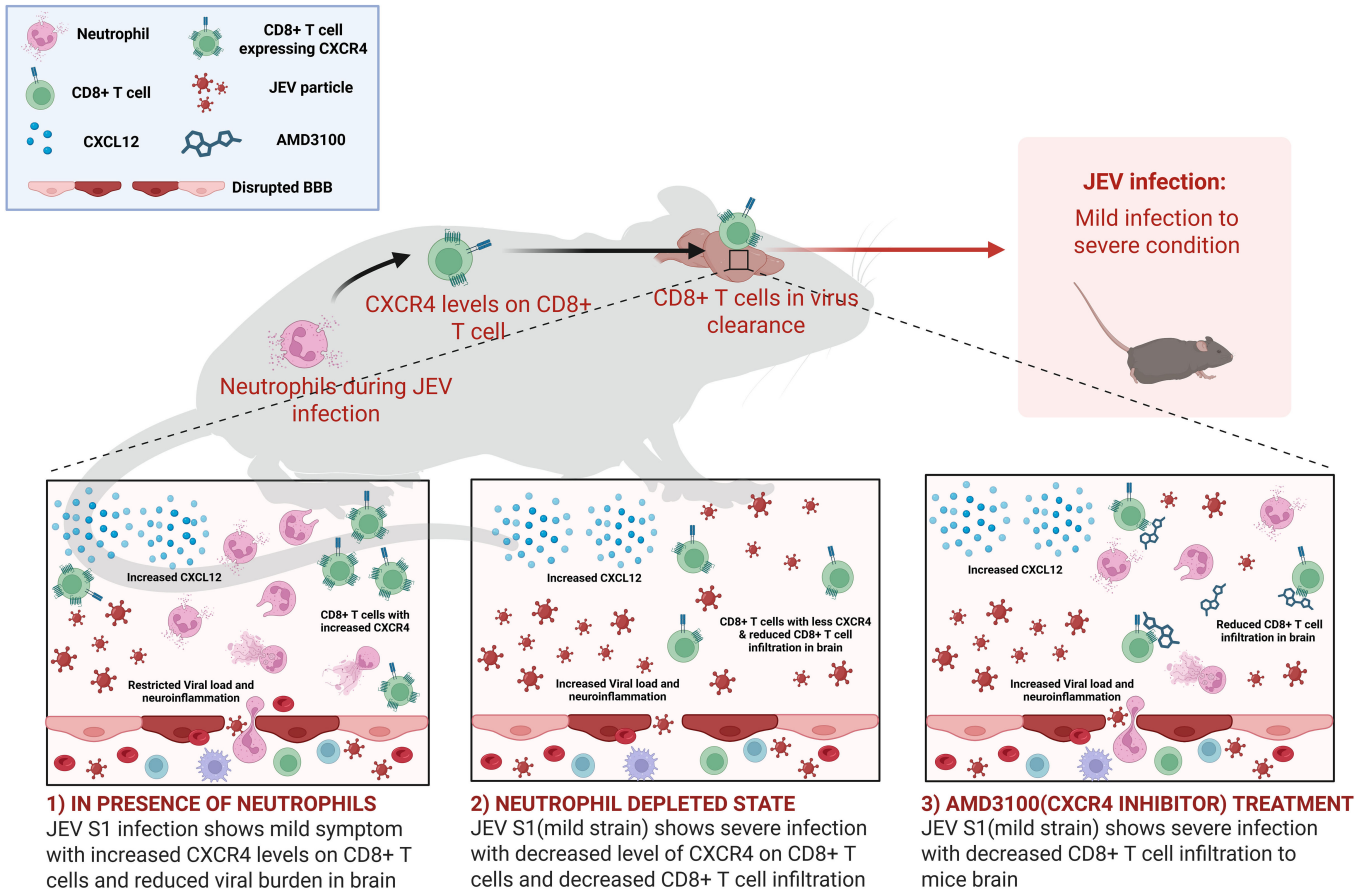
Schematic diagram representing the impact of neutrophil depletion and CXCR4 inhibition by AMD3100 on immune cell infiltration, viral replication and disease outcome.

In conclusion, we have used antibody-mediated depletion of Ly6G^+^ cells to investigate the role of neutrophils during JEV infection in mice. We demonstrate that neutrophils play a role in limiting disease severity following infection of mice with strains of mild virulence (JEV-S1), whereas they are not critical in controlling infection with virulent JEV-S3. Although neutrophil depletion did not affect CXCL12 levels in the brain, neutrophil depletion is associated with reduced CXCR4 expression on CD8 cells in the blood and brain. However, treatment of JEV-S1-infected mice with Anti-mouse CD8a antibody was associated with delayed encephalitis symptoms and enhanced virus replication, indicating that CD8^+^ T cells and neutrophils limited disease severity during mild JEV infections (**Figure 13**). Together, our study sheds new light on the role of neutrophils in the pathogenic mechanisms of JEV encephalitis and highlights the importance of neutrophils and CD8 cells, which are associated with disease outcomes.

## Data Availability

The raw data supporting the conclusions of this article will be made available by the authors, without undue reservation.
